# Differential expression of ion channel coding genes in the endometrium of women experiencing recurrent implantation failures

**DOI:** 10.1038/s41598-024-70778-9

**Published:** 2024-08-27

**Authors:** Bahar Davoodi Nik, Danial Hashemi Karoii, Raha Favaedi, Fariba Ramazanali, Maryam Jahangiri, Bahar Movaghar, Maryam Shahhoseini

**Affiliations:** 1 Department of Genetics, Faculty of Basic Sciences and Advanced Technologies in Biology, University of Science and Culture, Tehran, Iran; 2 Department of Cell and Molecular Biology, School of Biology, College of Science, University of Tehran, Tehran, Iran; 3 Department of Genetics, Reproductive Biomedicine Research Centre, Royan Institute for Reproductive Biomedicine, ACECR, No. 12, Hafez St., Banihashem Sq, Resalat Ave., P.O. Box: 19395-4644, Tehran, Iran; 4 Department of Endocrinology and Female Infertility, Royan Institute for Reproductive Biomedicine, ACECR, Tehran, Iran; 5 Department of Embryology, Reproductive Biomedicine Research Centre, Royan Institute for Reproductive Biomedicine, ACECR, No. 12, Hafez St., Banihashem Sq, Resalat Ave., P.O. Box: 19395-4644, Tehran, Iran

**Keywords:** Ion channel, Gene ontology, Endometrium, Recurrent implantation failure, ChIP real-time PCR, Computational biology and bioinformatics, Developmental biology, Genetics, Molecular medicine

## Abstract

Our study probed the differences in ion channel gene expression in the endometrium of women with Recurrent Implantation Failure (RIF) compared to fertile women. We analyzed the relative expression of genes coding for T-type Ca2+, ENaC, CFTR, and KCNQ1 channels in endometrial samples from 20 RIF-affected and 10 control women, aged 22–35, via microarray analysis and quantitative real-time PCR. Additionally, we examined DNA methylation in the regulatory region of KCNQ1 using ChIP real-time PCR. The bioinformatics component of our research included Gene Ontology analysis, protein–protein interaction networks, and signaling pathway mapping to identify key biological processes and pathways implicated in RIF. This led to the discovery of significant alterations in the expression of ion channel genes in RIF women’s endometrium, most notably an overexpression of CFTR and reduced expression of SCNN1A, SCNN1B, SCNN1G, CACNA1H, and KCNQ1. A higher DNA methylation level of KCNQ1’s regulatory region was also observed in RIF patients. Gene-set enrichment analysis highlighted a significant presence of genes involved with ion transport and membrane potential regulation, particularly in sodium and calcium channel complexes, which are vital for cation movement across cell membranes. Genes were also enriched in broader ion channel and transmembrane transporter complexes, underscoring their potential extensive role in cellular ion homeostasis and signaling. These findings suggest a potential involvement of ion channels in the pathology of implantation failure, offering new insights into the mechanisms behind RIF and possible therapeutic targets.

## Introduction

Recurrent implantation failure (RIF) is a clinical complication that physicians encounter during in-vitro fertilization (IVF)^1^. RIF is defined by the inability to achieve a clinical pregnancy after the transfer of at least five high-quality embryos in at least two or more IVF cycles, according to the European Society of Human Reproduction and Embryology (ESHRE) guidelines^2^. This condition suggests that the embryo does not implant successfully in the uterine lining. In contrast, recurrent miscarriage is characterized by the loss of two or more clinical pregnancies, which means the embryo initially implants and starts to develop but is subsequently lost. These conditions require different diagnostic and therapeutic approaches due to their distinct natures and underlying causes^3^. RIF could be related to the type and quality of clinical action and also the population of cases studied. Implantation failure may also be related to many factors that are classified into three categories: reduced endometrial receptivity, embryonic defects and multifactorial causes^4^. RIF cases may also be idiopathic, which means there is no identifiable problem in parents^5^. Many genes and proteins that are expressed during the window of implantation have been identified in earlier studies^6,7^. Up-regulated genes that related to endometrial receptivity are recognized to adjust cytokine-cytokine interaction pathways, complement and coagulation cascades, extracellular membrane receptor interaction, and inhibition of matrix-metalloproteinase pathway^8–11^. Expression of these genes prior to the window of implantation may prepare the endometrium in terms of structural and functional for embryo attachment. However, the ways to control such a complex set of genes involved in the regulation of endometrial receptivity remains largely unknown^7^. Generally, 62.25% of the receptivity-associated genes that up-regulated during window of implantation, encode the extracellular and plasma membrane proteins^12^. These proteins may be important in embryo adhesion and attendant signal transmission pathways. The important roles of ion channels in indirect activation of signal pathways that regulate endometrial receptivity and embryo implantation have been emphasized in recent studies^7,13–15^.

Endometrial ion channels play a major role in endometrial receptivity regulation and embryo implantation^16^. Many ion channels are involved in embryo implantation due to their role in the regulation of the volume of intra uterine fluid, decidualization and indirectly expression of the implantation associated genes^17^. Abnormal expression or action of ion channels in the endometrium can lead to disruption of endometrial receptivity and embryo implantation failure. To date, more than 14 types of ion channels have been identified in human endometrium, including Epithelial Na+ Channel (ENaC), Cystic Fibrosis Transmembrane Conductance Regulator (CFTR) and various types of calcium and potassium channels^18–20^.

Uterine secretion absorption during pre-implantation phase is an innate phenomenon in many species that enables the embryo to stay near the uterine lumen before the penetration and implantation processes and to maintain its connection with the uterine endometrium^21^. Earlier studies have shown that ENaC is necessary to absorb uterine fluid and is thought to be responsible for decreasing uterine fluid during the pre-implantation phase. In recent years, the essential role of ENaC in the onset of embryo implantation has come to be recognized^17^. It has been shown that ENaC is activated by a protease that is released from the embryo, resulting a series of events occurring in endometrial epithelial cells including membrane depolarization events, activation of the voltage-sensitive Ca2+ channels, regulation of Cyclooxygenase type 2 (COX2) and the release of Prostaglandin E2 (PGE2), leading to the decidualization of stromal cells, which is prerequisite for successful embryo implantation^22,23^. Ruan et al. demonstrated that blocking of the epithelial sodium channel or knocking down of this gene in mouse uterus leads to embryo implantation failure^22^. The CFTR channel is an intermediate flow of Cl- and conducts the movement of water into the lumen and is essential for the secretion of the fluid of the epithelial cells^24,25^. It has been shown that on the third day after mating, the expression level of CFTR is increased but during implantation, it is reduced or even not expressed^26^. Shreds of evidence suggest that dynamic collaboration between CFTR and ENaC have an important role in regulating the volume of intraluminal fluid and to ensure blastocyst remaining^27^. In 2000, Chan et al. showed that the expression of ENaC has an inhibitory effect on CFTR in rat endometrium^28^.

T-type calcium channels known as Low Voltage-Activates (LVA) are expressed throughout the body including in the nervous system, heart, kidneys, smooth muscles, sperm, many endocrine organs and uterine endometrium^29–32^. Alpha-1 subunit of T-type calcium channel is the largest subunit of this channel and includes the voltage sensor section, pore and the regulatory area that are identified as a target by secondary messengers, drug substances and toxins^33^. Alpha-1 subunit of this subfamily is encoded by three genes—CACNA1G, CACNA1H, and CACNA1I^34^. Calcium ions play an important role in many cellular signaling pathways and cell adhesion regulation, which are essential for physiological processes of endometrial epithelial cell transformation and decidualization of stromal cells during embryonic implantation^35^. The complicated cascade reactions guide the adhesion between trophoblast and uterine epithelium^36^. Changes in the intracellular Ca2+ concentration as second messenger in endometrial epithelial cells may play a major role in embryo implantation^37^. In 2005, Liu et al. described the effect of oxytocin hormone on the T-type calcium channel in human uterine stromal cells and concluded that inhibition of the T-type calcium channel by oxytocin in stromal cells could be an important signal for the reconfiguration of the uterus during pregnancy^38^.

K+ channels have been found in endometrial epithelial cells and have an important role in the transmission of electrolytes in the uterus^7^. Studies have also shown that Na+ absorption and Cl- secretion depend on K+ activity in basal cells^39^. One of the known potassium channels in the endometrium is the KCNQ1. Its encoding gene (KCNQ1) is an imprinted gene whose expression is regulated by epigenetic processes. A non-coding RNA suppresses its paternal allele and as a result, the maternal allele of the KCNQ1 gene is expressed^40^. Considering the key role of endometrial ion channels in pre-implantation stage and their essential function for embryo implantation, this study evaluated the expression of ENaC, CFTR, T-type Ca2+ channels and KCNQ1 in the endometrium of women with recurrent implantation failure during the window of implantation compared to endometrium of the fertile oocyte donors during the implantation window^41^. Ion channels potentially play a pivotal role in the pathophysiology of RIF. Nevertheless, the necessity and precise function of these channels in the disease’s progression remain ambiguous. Previous research has primarily focused on the association between ion channel activity and various stages of RIF in animal models, such as rats. Moreover, there is a paucity of studies examining the expression of ion channels in the endometrial tissue. In this experimental study, we conducted a comprehensive investigation into the expression profiles of ion channels in RIF. To substantiate our findings, we employed advanced bioinformatics techniques, including microarray analyses and methylation database assessments. This integrative approach aims to provide a deeper understanding of the molecular underpinnings of RIF and the potential therapeutic implications of ion channels in its management.

## Material and methods

All methods performed in this study were in accordance with the relevant guidelines and regulations.

### Data collection of microarray datasets

This study systematically aggregated microarray datasets from the National Center for Biotechnology Information’s public repository (available at NCBI GEO: https://www.ncbi.nlm.nih.gov/geo/). Specifically, it incorporated four datasets pertinent to Recurrent Implantation Failure (RIF): GSE205398^42^ and GSE188409^43^, focusing on protein coding, and GSE121219^44^, emphasizing miRNA coding. Dataset GSE121219 encompasses endometrial samples from five RIF patients and five fertile controls during the Window of Implantation (LH+ 7), while GSE205398 and GSE205398 include samples from six and eight RIF patients, respectively, alongside a comparable number of fertile controls, collected during the mid-secretory phase**.**

### Microarray differential gene expression analysis

The study merged the three expression matrices, ensuring alignment on common genes, and addressed batch effects using the “removeBatchEffect” function from the R Bioconductor package “limma” (version 3.52.4) (https://bioconductor.org/packages/release/bioc/html/limma.html)^45^. Principal Component Analysis (PCA) was employed to discern inter- and intra-group variances between RIF and control groups. The differential expression of genes was determined via the “limma” package, adhering to a statistical significance threshold of |log2FC|> 1.5 and an adjusted *p*-value < 0.05. Visualization tools, including volcano plots and heatmaps, were utilized, with an emphasis on the 15 genes showing the most significant differential expression.

### Sampling and study design

Endometrial biopsies were obtained from endometrial injury of 20 women with RIF between 22 and 35 years old on days 19th–24th of the menstrual cycle (window of implantation period). The size of the samples was between 50 and 100 mg obtained by pipelle. These patients had undergone at least 3 failed IVF/ICSI cycles after 5 good quality embryo transfer. In addition, there were no obvious justifications for embryo implantation failures in these participants, such as ovarian tumors, RIF, polyps, fibroids, uterine malformation or even male factors. In addition, endometrial biopsies were obtained from 10 volunteers 22–35-year-old fertile oocyte donors through the window of implantation, one cycle before ovarian stimulation as control group. None of the controls had received hormonal treatments for three months before the biopsy. Consent was obtained from patients according to local ethical approval guidelines and the study was approved by the Royan Institute. Each sample was divided into two parts: one of which was preserved in RNAlater at − 70°C for RNA extraction and the second part was frozen at − 70°C for chromatin immunoprecipitation assay (ChIP).

### Gene expression

Relative expression of three genes encoding α, β and γ subunits of ENaC that are called SCNN1A, SCNN1B and SCNN1G, CFTR, KCNQ1, and three genes of alpha-1 subunit of T-type calcium channel subfamily including CACNA1G, CACNA1H, CACNA1I in endometrial samples of RIF and non-stimulated women were compared quantitatively by real-time PCR. RNA was extracted from endometrial samples of RIF and fertile group by TRIzol reagent (Invitrogen, Carlsbad, CA, USA, cat#. 15,596-018), conformity the suppliers’ protocol.

After DNase-I (TAKARA, cat #. K301BA) treatment to exclude DNA, purity, and concentration of RNA samples were determined using Nanodrop 2000 spectrophotometer (Thermo Scientific). Then complementary DNA was synthesized and cDNA was used for real-time PCR that was performed on 7500 Real-Time PCR System (AB Applied Biosystems, Carlsbad, CA, USA) using SYBR Green PCR master mix (Applied Biosystems), with designed primers and Taqman (gene expression assay ID: 4,448,489). In this research, target genes were normalized by the GAPDH as a reference gene and measured by the ΔCT method.

$$ {\text{CT }}\left( {\text{target gene}} \right) \, - {\text{CT }}\left( {\text{reference gene}} \right) \, = \, \Delta {\text{CT}} $$

The resulting numbers were converted to 2 − ΔCT, and subsequent analyses were performed on the 2 − ΔCT numbers of each sample. Then, comparisons and needful statistical analysis were performed using SPSS software version 22. Independent-Samples T-test or Mann–Whitney U-test was used to compare mRNA expression levels in the RIF patients and control group.

### ChIP assay

The presence of the DNA methylation levels in the regulatory region of KCNQ1 was evaluated using the ChIP real-time PCR technique by MeCP2 antibody through the following steps: cell fixation and crosslinking, sonication of lysate to shear DNA, immunoprecipitation, elution and reversal of cross-links. Immunoprecipitated DNA was quantified by real-time PCR on a 7500 Real-Time PCR System (AB Applied Biosystems). Statistical analysis used Independent-Samples T-test using the SPSS software version 22 to compare DNA methylation levels in the RIF patients and control group.

### Constructing and analyzing protein–protein interaction networks

To illuminate the functional implications of differentially expressed genes (DEGs) within a biological context, an intricate protein–protein interaction (PPI)^46^ network was meticulously formulated. This network serves as a scaffold to elucidate the complex web of interactions among the DEGs, thereby providing insight into their collective biological roles. The network’s construction was facilitated by leveraging the comprehensive STRING (https://string-db.org/)^47^ database, renowned for its extensive repository of known and predicted protein–protein interactions. Alongside, Cytoscape^9–11,15,20,30–32,48–52^ software, version 3.7.2, was utilized for its advanced graphical capabilities, allowing for intricate visualization and analysis of the PPI networks. To refine the network to its most influential components, the Molecular Complex Detection (MCODE) algorithm was harnessed as a Cytoscape plugin. This algorithm is adept at identifying densely connected regions within the PPI network, suggesting the formation of functional modules or clusters of hub genes. These modules often represent significant biological processes and are crucial for understanding the underlying molecular mechanisms.

The criteria set forth for module selection within the network were rigorously defined to ensure the extraction of relevant and robust interactions. A degree cutoff was instituted at a baseline of two connections to other nodes to filter out isolated or less significant proteins. Similarly, a node score cutoff was established at two, emphasizing proteins with a higher influence on the network’s topology. The K-core parameter was applied to ensure that each module contained a core inter-connected structure, with a minimum required connectivity degree of two. This ensures that the modules identified are not only interconnected but also resilient to random disconnections, reflecting potential stability in biological systems. Additionally, a maximal search depth of 100 was determined, which acts as a boundary for the distance from the seed node that MCODE^53^ could search for additional cluster members. This parameter is pivotal in balancing the breadth of the search without diluting the significance of the connections identified.

Upon the successful delineation of significant modules, the Kyoto Encyclopedia of Genes and Genomes (KEGG)^54^ pathway enrichment analysis was conducted through the online DAVID^55^ bioinformatics database. This analysis is integral as it associates the hub genes within the modules to known biological pathways, thereby facilitating a deeper understanding of their functional interplay and their potential impact on cellular processes. The enrichment analysis thus aids in discerning the biological pathways that are over-represented in the PPI network, providing valuable insights into the molecular etiology of the condition under study. Through such a comprehensive analytical approach, the PPI network analysis not only identifies key molecular players but also elucidates their dynamic interactions, thereby contributing to a more nuanced understanding of cellular functioning.

### GO and pathways enrichment analysis

In the domain of functional genomics, Gene Ontology (GO) analysis is an established method for categorizing genes into a structured network of defined terms spanning three primary ontologies: Biological Processes (BP), which elucidates the roles of genes within biological sequences of events; Cellular Components (CC), which describes the gene products’ localization within cellular structures; and Molecular Functions (MF), which specifies the biochemical activities at the molecular level. Concomitantly, the KEGG (https://www.genome.jp/kegg/) stands as a pivotal database that amalgamates genomic, chemical, and systemic functional information. It offers a macroscopic view of the molecular interaction and reaction networks that underpin cellular functions and organismal systems.

To facilitate the enrichment analysis of putative differentially expressed genes (DEGs), the R package “clusterProfiler”^56^ is utilized. This tool is adept at integrating high-throughput omics data and provides a comprehensive suite of bioinformatics analyses, including GO enrichment and KEGG pathway mapping. It is designed to identify and visualize statistically overrepresented pathways and interaction networks in a set of DEGs. Further enriching the analytical depth, Reactome3 is also employed as a pathway database that models human biological pathways, offering a detailed perspective on pathway knowledge that complements the data obtained from KEGG. Reactome^56^ provides an alternative lens through which the biological significance of gene sets can be discerned.

In the execution of these analyses, statistical rigor is maintained by adopting an adjusted p-value less than 0.05 as the cut-off criterion. This stringent threshold ensures that the likelihood of identifying false positives due to multiple testing is minimized, thus conferring greater confidence in the biological relevance of the findings. The p-value adjustment, often achieved through methods like the Benjamini–Hochberg procedure, is imperative in high-throughput genomic studies to control for the false discovery rate. This comprehensive analytical framework facilitates a nuanced understanding of the biological themes and pathways that are significantly associated with the DEGs under study, enabling researchers to draw meaningful inferences about the underlying biological processes and their potential implications in the context of health and disease.

### Construction of miRNA-centric gene regulatory networks

The ENCORI platform, also known as the Encyclopedia of RNA Interactomes, is a comprehensive, freely accessible resource dedicated to the study of interactions between miRNAs that we get significant miRNA in GSE121219 (miRNA coding) and our targeted genes (available at http://starbase.sysu.edu.cn/; version 3.0). ENCORI integrates data from eight authoritative miRNA-target prediction repositories, namely PITA, miRmap, microT, miRanda^57^ and TargetScan^58^. For the purposes of this research, miRNAs were designated as the targeted regulators of central genes if they were recognized by at least two of the following prediction databases: miRanda, PITA, PicTar, and TargetScan.

### Ethics approval and consent to participate

The study protocol was approved by Ethical Committee of Royan Institute for Reproductive Biomedicine (IR.ACECR.ROYAN.REC.1395.041). Informed consent was obtained from all the participants for being included in the study.

## Results

### Rectified variance in microarray datasets and identification of differential mRNA expression

**Fig. 1:**
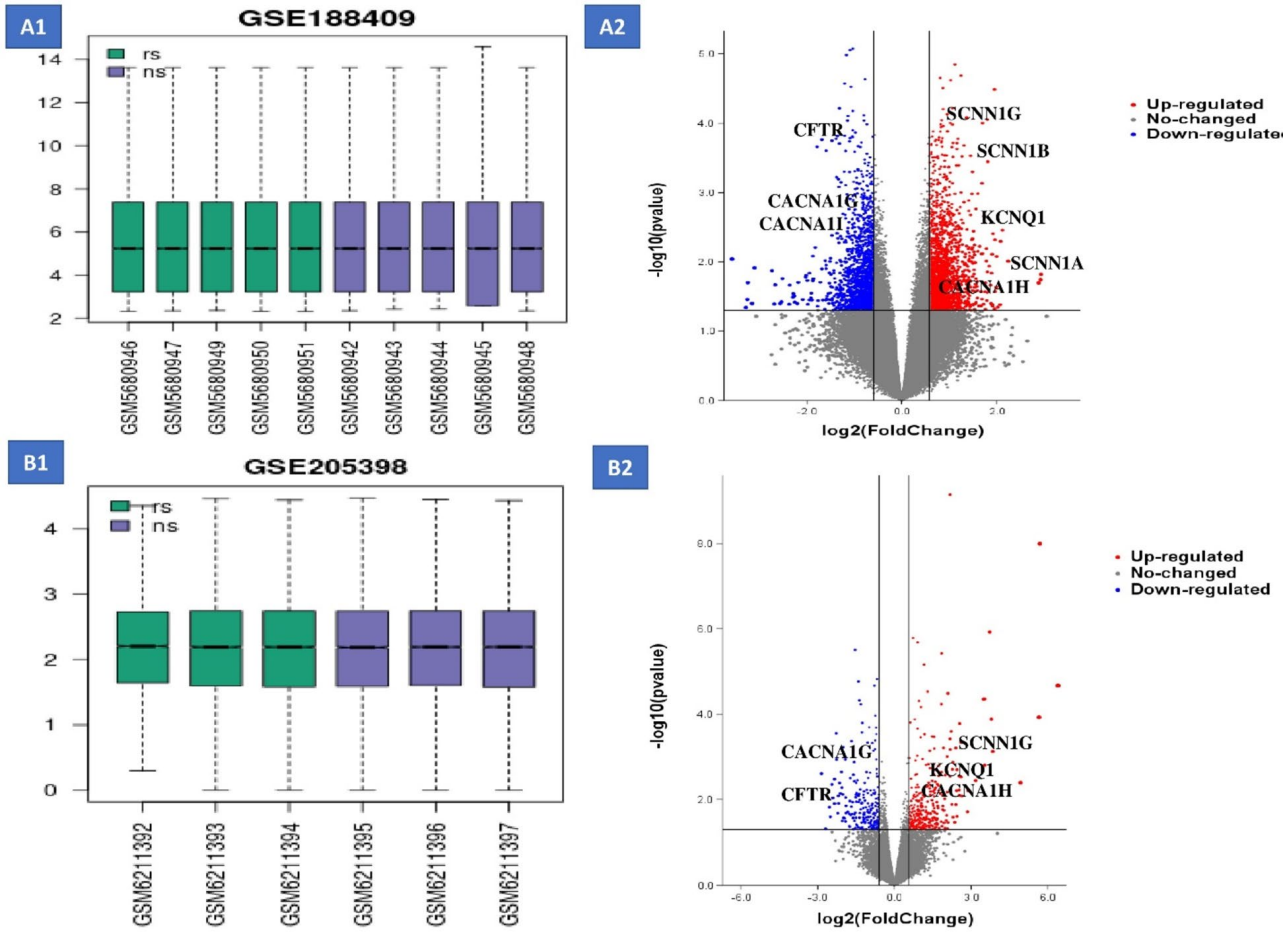
Microarray gene expression related to RIF. (**A1**) boxplot in GSE188409 that related to gene expression in RIF, (**A2**) boxplot in GSE188409 that related to gene expression in RIF, (**B1**) boxplot in GSE205398 that related to gene expression in RIF and (**B2**) volcano plot in GSE205398 that related to gene expression in RIF. In the volcano plot, the blue dots represent genes that are downregulated, while the red dots correspond to genes that are upregulated.

The integration of three distinct microarray datasets, post-rectification for batch effects, revealed a marked reduction and normalization in variance across these datasets. The principal component analysis (PCA) demonstrated minimal intragroup variance within the Recurrent Implantation Failure (RIF) and control groups, whereas the intergroup variance was found to be significant. This distinct pattern underscores the potential for downstream differential gene expression analysis. We identified 279 differentially expressed genes between the RIF and control groups, which included 141 up-regulated and 138 down-regulated genes (referenced from GSE188409 and GSE205398). The differential expression of these genes, characterized by specific log-fold changes (log2FC) and *p*-values, is depicted in the accompanying volcano plot. This evidence strongly indicates significant genetic expression alterations in the microarray datasets (Fig. 1 and supplementary 1).

### Gene expression analysis

**Fig. 2:**
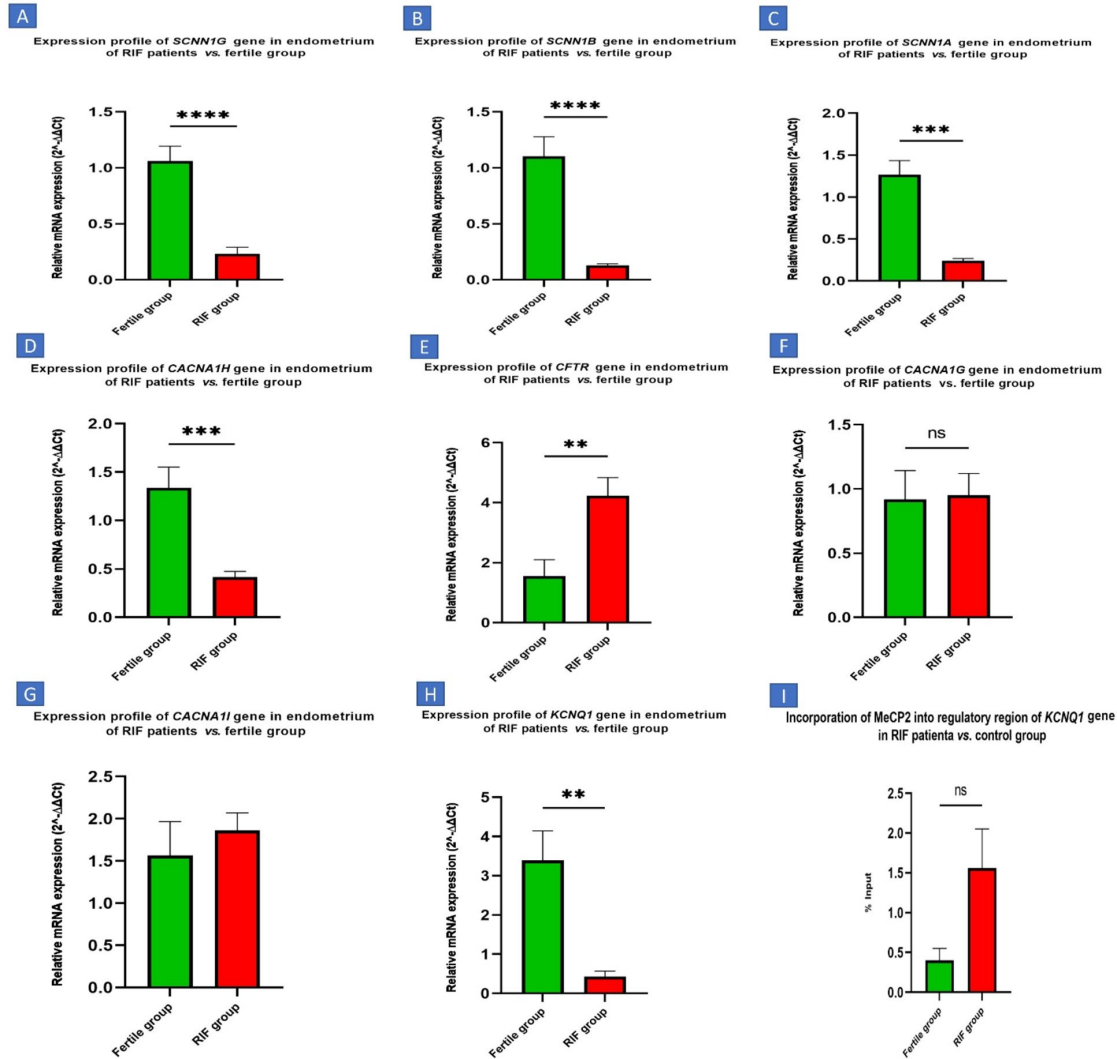
Ion channel gene expression (**A**) SCNN1G, (**B**) SCNN1B, (**C**) SCNN1A, (**D**) SCNN11H, (**E**) CFTR, (**F**) CACNA1G and (**G**) CACNA1G and (**H**) KCNQ1 in RIF and Fertile women during implantation (*P* ≤ 0.01**, *P* ≤ 0.001***). (**I**) a higher level of MeCP2 in the regulatory region of KCNQ1 in RIF.

In order to determine the genes expression level of T-type Ca2+ channel in the endometrium, three alpha-1 subunits of this subfamily, viz., CACNA1G, CACNA1H, and CACNA1I were evaluated. CACNA1G and CACNA1I expressions in endometrial tissue of RIF women were relatively increased, but the changes were not significant. On the other hand, as shown in Fig. 1, the CACNA1H in the endometrium of RIF women showed a significant decrease compared to the expression of this gene in the endometrial tissue of fertile women with *p*Value ≤ 0.001. As shown in the diagram, significant over-expression of CFTR was observed in endometrial tissue of RIF women compared to the control group (*P* ≤ 0.01). Expression levels of SCNN1A, SCNN1B and SCNN1G genes that code three subunits of ENaC, are significantly lower in the endometrium of RIF women than in the control group (*P* ≤ 0.001). By examining the expression of the KCNQ1 in the collected samples, it was found that KCNQ1 gene expression decreased significantly in endometrial tissue of RIF women compared to control group women (*P* ≤ 0. 05) (Fig. 2).

### Chromatin immunoprecipitation

Epigenetic changes in the regulatory region of KCNQ1 were investigated using ChIP technique by MeCP2 antibody following expression assessment in control and experiment groups. The results showed a higher level of MeCP2 in the regulatory region of KCNQ1 in RIF patients than the control group (*P* ≤ 0. 05) (Fig. 2).

### Construction of PPI network

**Fig. 3:**
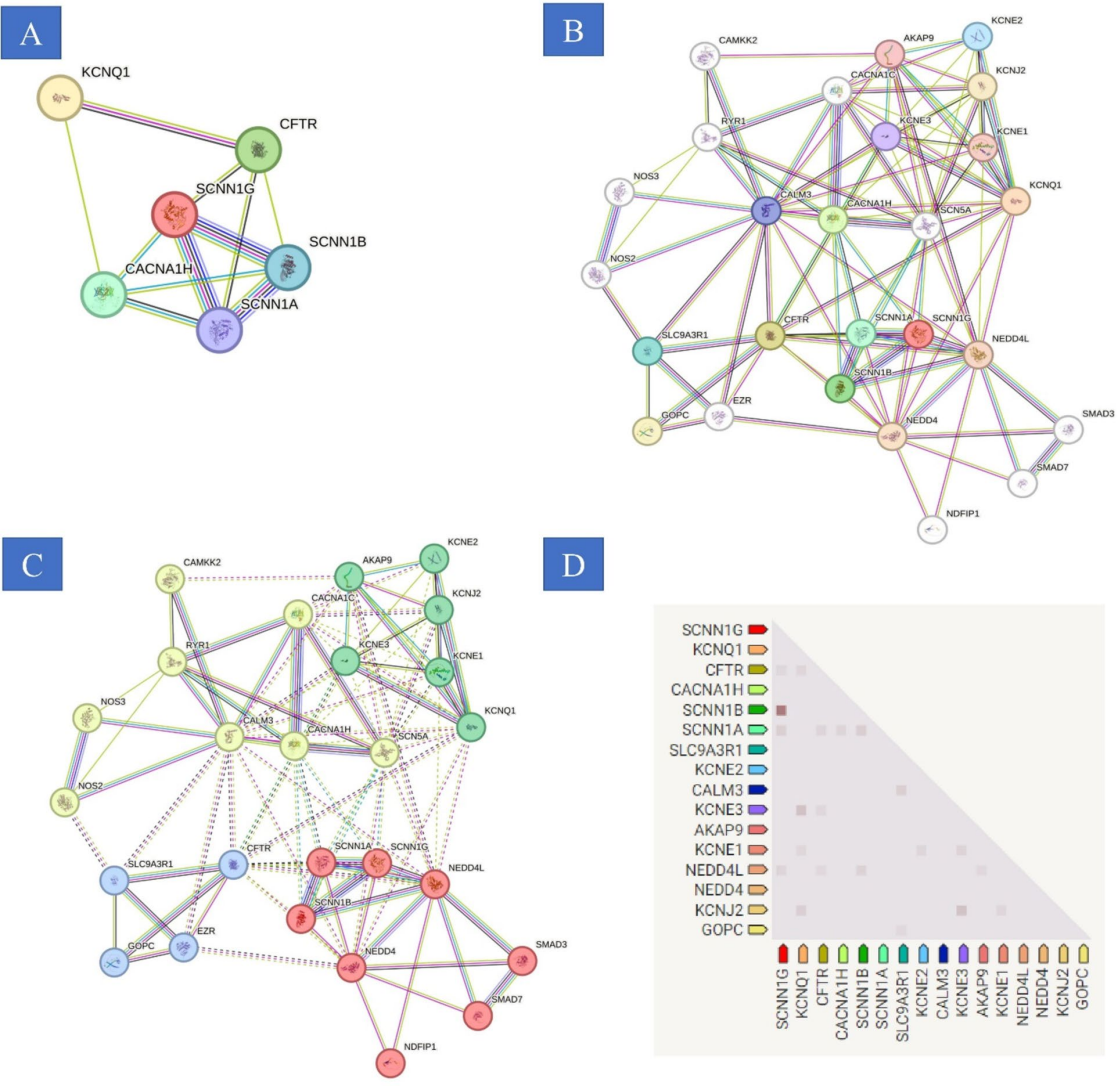
PPI network. (**A**) Utilizing the STRING database, a comprehensive PPI network was meticulously assembled, encompassing all six DEGs, with an established interaction score threshold of 0.4 to ensure specificity. (**B**) In the context of temporomandibular joint internal derangement (TMJD), the curated PPI network elucidated via STRING identified six genes—CFTR, SCNN1A, SCNN1B, SCNN1G, CACNA1H, and KCNQ1—as central nodes, distinguished by their substantial connectivity within the network.

The six principal genes were incorporated into the String database to establish Protein–Protein Interaction (PPI) networks, adhering to a minimum threshold of 0.4 for interaction scores. The visualization of these interactions was facilitated through the application of Cytoscape software. Utilizing the Degree algorithm, a subset of the ten most significant genes was discerned, including CFTR, SCNN1A, SCNN1B, SCNN1G, CACNA1H, and KCNQ1. This selection process revealed a substantial correlation amongst the upregulated and downregulated differentially expressed genes (DEGs). Further analysis indicated a pronounced positive co-expression among the genes KCNE1B, KCNJ1, NEDD4L, SCN1B, SCN2B, SCN3B, SCN4A, SCN4B, SCN7A, SCNN1D, SLC12A3, TRPM4, WNK1, WNK3, and WNK4 (Fig. 3 and supplementary 2).

### Analysis of biological functions and signaling pathways

**Fig. 4:**
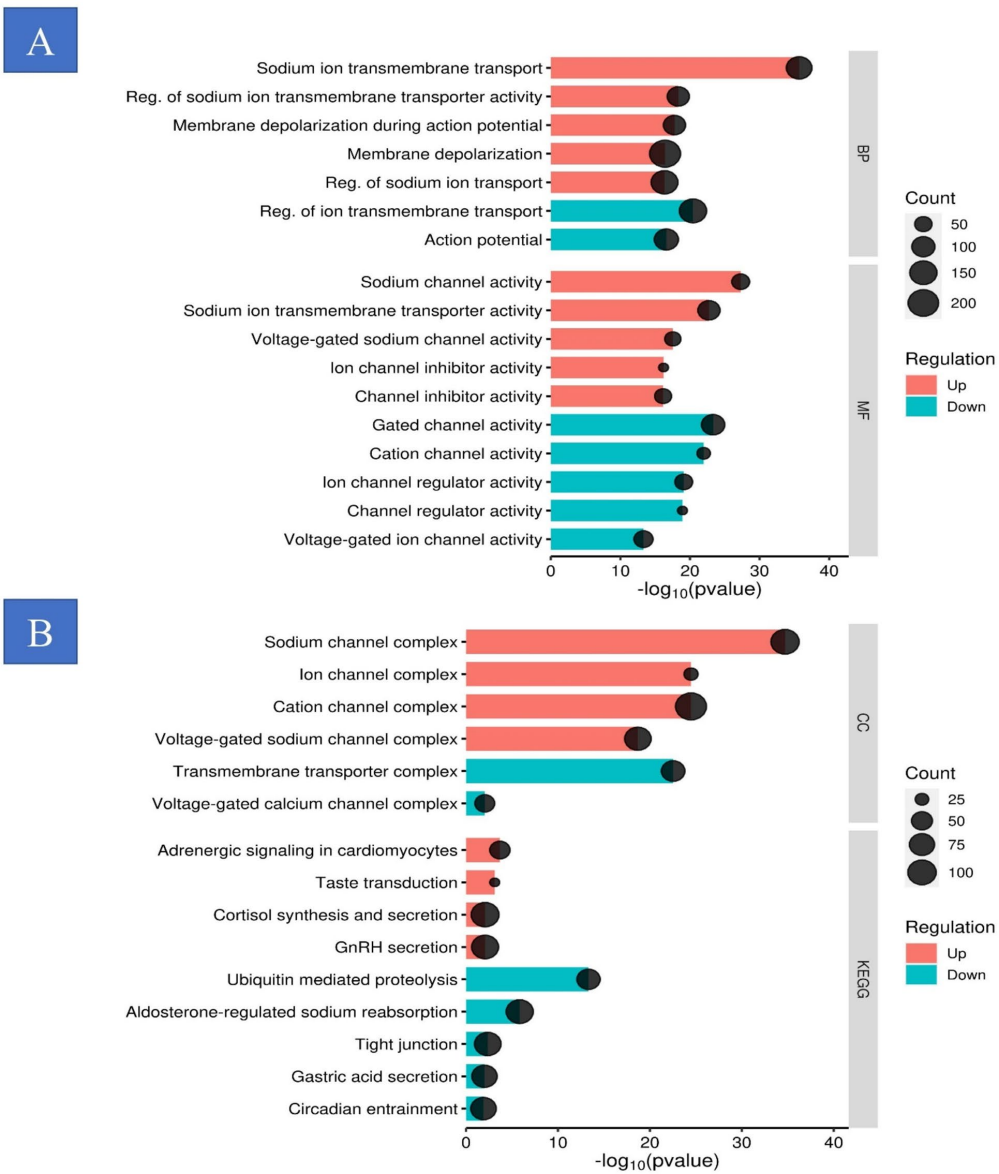
GO analysis. (**A**) Cellular component (CC), (**B**) biological process (BP), (**C**) molecular function (MF), and (**D**) Kyoto Encyclopedia of Genes and Genomes (KEGG) enrichment analysis. GO, Gene Ontology.

**Fig. 5:**
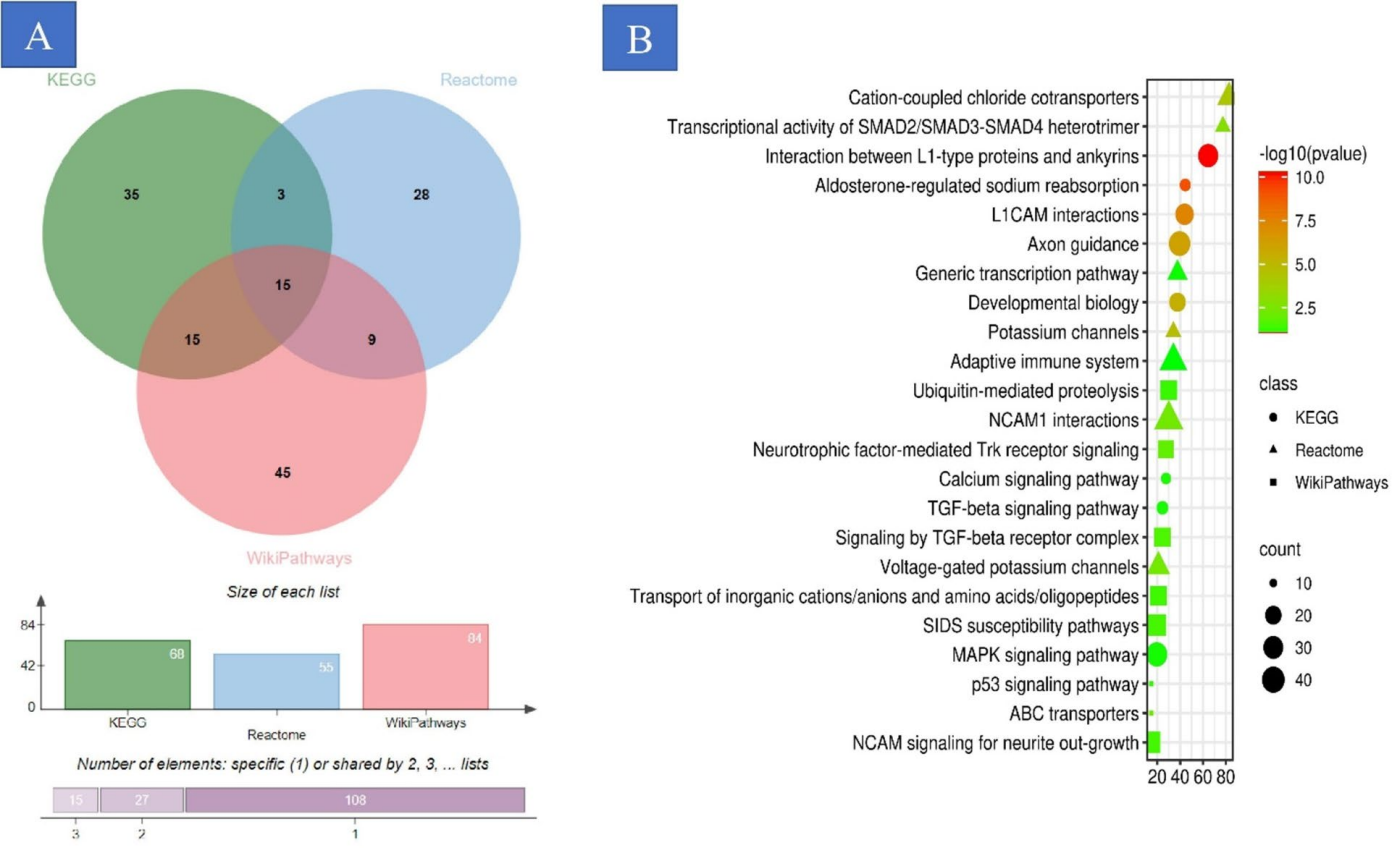
Reactome, KEEG and wikipathways show that up/ down-regulation genes can contribute to different types of aldosterone-regulated sodium reabsorption, gonadotropin-releasing hormone secretion, cortisol synthesis and secretion.

The gene ontology (GO) enrichment analysis, graphically represented through a dot plot, elucidated that genes pertaining to the biological process (BP) category were predominantly associated with functions of the voltage-gated sodium system, which include the Sodium channel complex, voltage-gated sodium channel complex, cation channel complex, ion channel complex, voltage-gated calcium channel complex, and transmembrane transporter complex (Fig. 4A,B). Concurrently, cellular component (CC) category genes demonstrated a pronounced association with similar complexes, reinforcing the specificity of these genes to their functional assemblies. In the molecular function (MF) category, there was a distinct association with activities relating to voltage-gated ion channels, including voltage-gated cation channel activity and metal ion transmembrane transport, indicating their pivotal role in cellular electrophysiology (Fig. 4C,D and supplementary 3). Moreover, the KEGG pathway enrichment analysis underscored a significant correlation with pathways governing aldosterone-regulated sodium reabsorption, gonadotropin-releasing hormone (GnRH) secretion, cortisol synthesis and secretion, and adrenergic signaling in cardiomyocytes, highlighting their potential impact on physiological processes (Fig. 5 and supplementary 4).

### miRNA-mRNA network

**Fig. 6:**
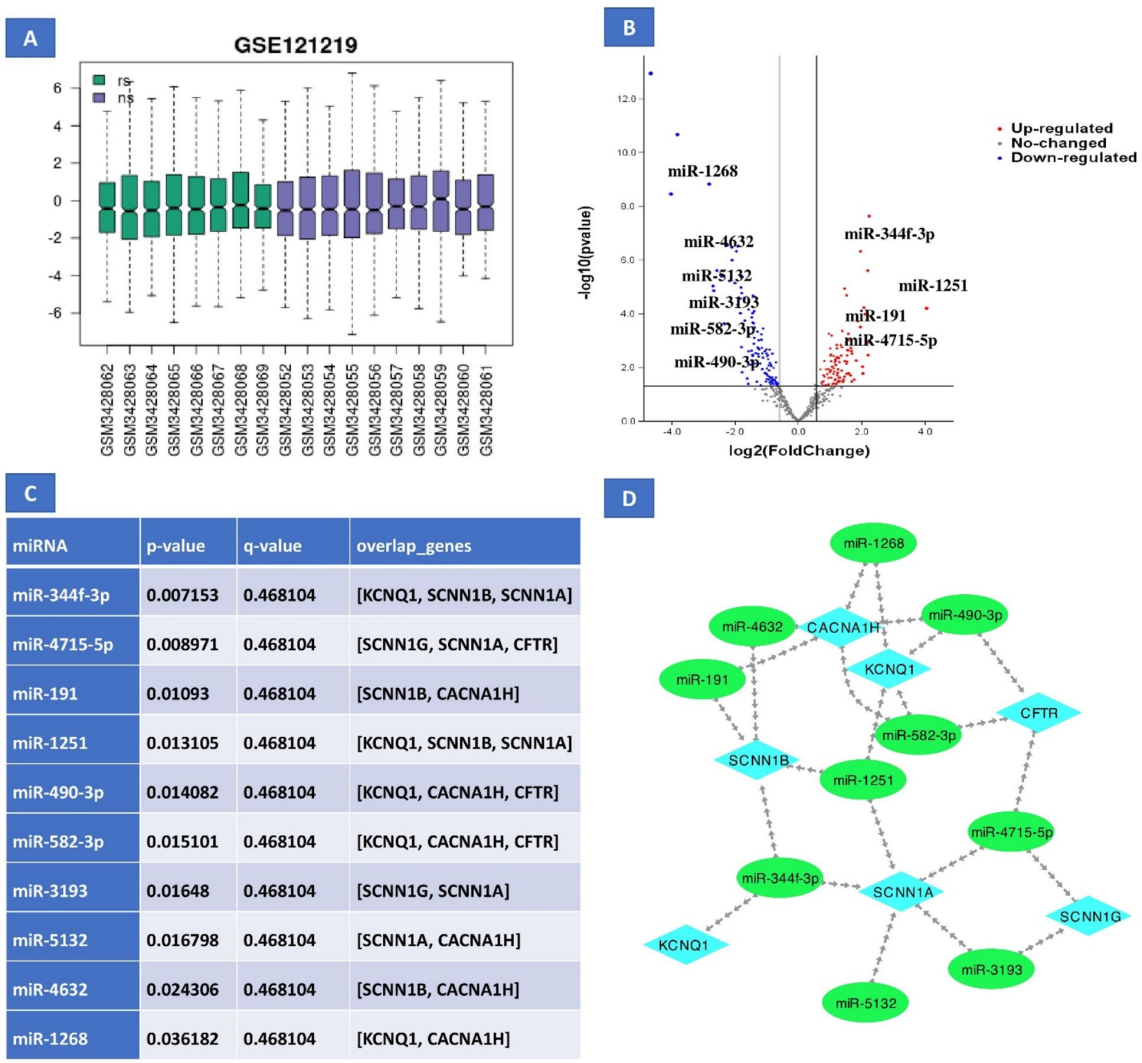
Microarray miRNA expression related to RIF and interaction with target genes. (**A**) boxplot in GSE121219 that related to miRNA expression in RIF and (**B**) volcano plot in GSE121219 that related to gene expression in RIF. (**C**) miRNA-mRNA interaction with p-value and (**D**) miRNA-mRNA network. In the volcano plot, the blue dots represent genes that are downregulated, while the red dots correspond to genes that are upregulated.

Four different databases (Tragetscan, PITA, PicTar, and miRanda) from ENCORI were used for the identification of miRNAs targeted which we get it in GSE121219 by the hub genes. The miRNA was considered as targeted miRNA of that particular hub genes, if the resulted miRNA were present in at least 2 databases (Tragetscan, PITA, PicTar, and miRanda) (Supplementary file 2). Using degree method in cytoHubba plugin, the topmost 10 molecules were selected. Moreover miR-344f.-3p (*p*-value = 0.007153) and miR-4715-5p (overlap genes: KCNQ1, SCNN1B, SCNN1A, *p*-value = 0.008971) were the top miRNAs that considered to be targeted most hub genes (Fig. 6 and supplementary 5).

## Discussion

The study of RIF in women has unveiled intriguing insights into the role of ion channel gene expression within the endometrium. All participants in the research were within the prime reproductive age bracket of 22–35 years, a detail that underscores the pertinence of the findings to the population most affected by RIF. The study’s findings indicated a dysregulated expression of genes responsible for encoding ion channels, particularly noting a significant over-expression of the CFTR gene in the endometrial tissue of RIF patients when compared to the control group. This suggests that CFTR could be playing a pivotal role in the endometrial environment, potentially affecting implantation success. Moreover, the study highlighted a pronounced decrease in the expression levels of other crucial ion channel genes such as SCNN1A, SCNN1B, SCNN1G, and CACNA1H in the endometrium of women experiencing RIF. The altered expression patterns of these genes, which are essential for sodium and calcium ion transport, were statistically significant and point towards a potential disruption of ionic balances that are critical during the window of implantation. The decreased expression of the KCNQ1 gene, along with a higher presence of MeCP2 in its regulatory region, further implicates the intricate network of ion channels in the endometrial receptivity process.

Integrant bioinformatics emerges as a pivotal tool in understanding the complexities of gene expression and regulation within the context of RIF. The utilization of bioinformatics allows for a comprehensive analysis of gene expression data, providing a clearer picture of the molecular interactions at play. The significant alteration in ion channel gene expression observed in RIF patients offers a new avenue of exploration, suggesting that these channels could be integral to creating a conducive environment for embryo implantation^17^. This research opens up potential pathways for therapeutic interventions and sheds light on the molecular underpinnings that may contribute to implantation failures, with bioinformatics serving as the backbone for these advancements. Reduced endometrial receptivity is responsible for about two-thirds of embryo implantation failures, while embryonic problems account for only one-third of the failures^59^. The endometrial function is directly regulated by factors involved in pH, oxidative stress and ion concentration to create an enabling environment for fertilization^60^. Uterine fluid tends to be alkaline following mating, which preserves sperm motility and survival while low pH prevents sperm activation and pregnancy^61^. This could be evidence for the effectiveness of pH and ion gradient in women fertility. Previous studies have shown that the calcium channel has an important role in decidualization process and the blockage of this channel is accompanied by a reduction in the decidualization^62^.

A study conducted in 2012 showed that knockdown or blocking of the calcium channel BK (Ca+ 2) not only reduces the embryo implantation in rats but also significantly reduces the expression of endometrial receptivity factors such as the leukemia inhibitory factor, integrin β3, Claudin4 and DKK-1 in the endometrium^63^. In the present study, expression assessment of the alpha-1 subunit of T-type Ca+ 2 channel in RIF and fertile woman showed that the expression of the CACNA1H is significantly lower in women with RIF than control^63^. This is in accordance with previous studies that indicate that the down-regulation of T-type Ca+ 2 channel can be affecting the embryo implantation by influencing on endometrial receptivity^64,65^. The interaction between CFTR and ENaC ion channels is suggested as the main cause for regulating the absorption of fluids and secretion of endometrium epithelial cells in the preimplantation period in women. A study in 2014 reported the expression level of CFTR and ENa-α genes in relation to early abortion in mice models^66^. It showed an increase in the expression of the CFTR gene and decrease of expression of ENa-α in decidual cells of women with early abortion. In our study, the expression pattern of CFTR and ENaC in RIF patients are similar to women with early abortion^67^. Endometrial samples of RIF women showed a significant increase in expression of the CFTR channel gene and also a substantial decrease in ENaC channel encoding genes in comparison with the control group during the window of implantation^68^. Increased activity of the CFTR channel and simultaneous reduction of the ENaC channel activity in the pre-implantation phase may lead to accumulation of uterine fluids and embryo implantation failure^68,69^. Endometrial thickness measurement is used as a reliable indicator to ensure successful implantation after IVF^70^. Some studies have linked the abnormal expression or function of the K+ channels with a thin endometrium and unsuccessful implantation. These data provide pieces of evidence for the important role of potassium channel in the proliferation of endometrium epithelial cells and the necessity of its proper expression for a successful pregnancy^19^. The evidences are confirmed by our findings that the KCNQ1 potassium channel encoding gene reduces, and there is an increase in the DNA methylation level of the regulatory region of this gene in the endometrium of women with RIF compared with fertile women^71,72^. Generally, ion channels crucial for the activation or inhibition of epithelial secretion. Many pathways and physiological processes lead to accurate regulation of their expression.

In addition to these findings, Rull et.al study focusing on recurrent miscarriage (RM) profiled whole-genome differential gene expression in RM placental tissue, aiming to identify predictive biomarkers for threatening miscarriage. This study discovered 30 differentially expressed transcripts in RM placentas, with significant increases in mRNA expression of TNF-related apoptosis-inducing ligand (TRAIL) and S100A8, which encodes for the inflammatory marker calprotectin (S100A8/A9). These findings were validated by RT-qPCR. Further, significantly higher maternal serum concentrations of soluble TRAIL (sTRAIL) were observed in RM events and in women who developed an unpredicted miscarriage compared to normal first trimester pregnancies. Elevated sTRAIL levels were also noted in tubal pregnancies. While maternal serum levels of calprotectin were not diagnostic or prognostic for early pregnancy failures, the study indicated that sTRAIL could serve as a potential predictive biomarker for early pregnancy complications^73^. Moreover, it is essential to consider the broader embryonal genomic landscape in the context of assisted reproductive technologies like IVF. Although chromosomal instability (CIN) is a common phenomenon in cleavage-stage embryogenesis following IVF, its rate in naturally conceived human embryos is unknown. CIN leads to mosaic embryos that contain a combination of genetically normal and abnormal cells and is significantly higher in in vitro-produced preimplantation embryos as compared to in vivo-conceived preimplantation embryos. A recent study profiled the genomic landscape of fetal and placental tissues postpartum from both IVF and naturally conceived children to investigate the prevalence and persistence of large genetic aberrations that probably arose from IVF-related CIN. The study demonstrated that CIN is not preserved at later stages of prenatal development and de novo numerical aberrations or large structural DNA imbalances occur at similar rates in IVF and naturally conceived live-born neonates. According to these data we aim to provide a more comprehensive understanding of the endometrial factors contributing to recurrent implantation failure^74^.

Several genes are implicated in the processes related to GnRH secretion and its influence on RIF. These genes can be involved in various aspects such as hormone regulation, inflammation, cell proliferation, and tissue response. GnRHR gene codes for the receptor for GnRH. Variations in this gene can affect how the body responds to GnRH, influencing the release of gonadotropins and subsequently the menstrual cycle and estrogen production. CYP19A1is an enzyme that is crucial for estrogen biosynthesis. In RIF, increased expression of the aromatase gene in ectopic endometrial tissue leads to higher local estrogen production, which can exacerbate the condition. The estrogen receptor alpha (ESR1) and beta (ESR2) are important in mediating the effects of estrogen. Alterations in these genes can influence the development and progression of RIF. Prostaglandin-Endoperoxide Synthase 2 (PTGS2/COX-2) gene is involved in the inflammatory response and is often found to be overexpressed in endometriotic lesions. It plays a role in pain and inflammation associated with RIF. VEGF (Vascular Endothelial Growth Factor) gene is key in angiogenesis, the formation of new blood vessels, which is crucial for the survival and growth of endometriotic tissues. HOX (Homeobox) genes are involved in the developmental processes and have been implicated in the pathogenesis of RIF. Abnormal expression of certain HOX genes may contribute to the ectopic growth of endometrial cells. MMPs (Matrix Metalloproteinases) are involved in the breakdown and remodeling of the extracellular matrix, which is a critical process in the invasion and implantation of endometriotic tissues. Our comprehensive bioinformatics analysis indicates that mutations in these genes are implicated in the pathogenesis of RIF, characterized by the aberrant proliferation and ectopic implantation of endometrial cells.

## Conclusion

Previous studies and our present study show that the effects of ion channels on endometrial receptivity and early stages of pregnancy are undeniable. It can be concluded that the abnormality of endometrial ion channel expression is a reason for endometrial impotency and implantation failure. Further studies on the role of ion channels in endometrial activity and accurate recognition of their function, can help in understanding inhibition or overexpression of these genes. Furthermore, the establishment of a suitable concentration of ions in the uterine fluid in the preimplantation stage, through control of these channels function may provide favorable conditions for embryo implantation.

### Supplementary Information

Supplementary Information 1.

Supplementary Information 2.

Supplementary Information 3.

Supplementary Information 4.

Supplementary Information 5.

## Data Availability

The analytical code employed in this study can be accessed at the following GitHub repository: https://github.com/DanialHashemiKaroii77/Analysis-of-Differential-Ion-Channel-Gene-Expression-Recurrent-Implantation-Failures.git.

## Abbreviations

RIF: Recurrent implantation failure
IVF: In-vitro fertilization
PPI: Protein-protein interaction
DEGs: Differentially expressed genes
ENaC: Epithelial Na+ Channel
CFTR: Cystic Fibrosis Transmembrane Conductance Regulator
GO: Gene ontology
