# Supplementary material for: Differential expression of ion channel coding genes in the endometrium of women experiencing recurrent implantation failures
Source: Sci Rep. 2024 Aug 27;14:19822. doi: 10.1038/s41598-024-70778-9 (PMC11349755; doi:10.1038/s41598-024-70778-9)
Supplement: Supplementary file 1 — Supplementary Information 1. [file 41598_2024_70778_MOESM1_ESM.docx]

| Sheet 1  **Significantly differentially gene expression in GSE188409** | | | | | |  |  |
| --- | --- | --- | --- | --- | --- | --- | --- |
| **ID** | **adj.P.Val** | **P.Value** | **t** | **B** | **logFC** | **ORF** | **SPOT_ID** |
| ASHGV40004847V5 | 0.165 | 3.26E-05 | 7.24 | 2.0662 | 1.95 | SCNN1A |  |
| ASHG19LNC1A100051630V5 | 0.185 | 8.87E-05 | 6.4 | 1.3663 | 1.8 | SCNN1B |  |
| ASHGV40053005V5 | 0.185 | 9.98E-05 | 6.31 | 1.2807 | 1.7 | G085208 |  |
| ASHG19LNC1A100032486V5 | 0.223 | 0.00036 | 5.34 | 0.3123 | 1.81 | AC005332.2 | |
| ASHGV40004848V5 | 0.27 | 0.000735 | 4.84 | -0.2546 | 1.69 | CCDC26 |  |
| ASHG19AP1B110823932V5 | 0.309 | 0.001036 | 4.61 | -0.5333 | 1.6 | TSPAN13 |  |
| ASHG19AP1B100206264V5 | 0.343 | 0.001725 | 4.28 | -0.9533 | 1.56 | FZD5 |  |
| ASHG19AP1B100007395V5 | 0.351 | 0.002201 | 4.12 | -1.1568 | 1.73 | LDHAL6B |  |
| ASHG19AP1B100083243V5 | 0.366 | 0.003493 | 3.83 | -1.5453 | 2.12 | GPR37 |  |
| ASHGV40030071V5 | 0.37 | 0.003615 | 3.81 | -1.5745 | 1.51 | G047908 |  |
| ASHG19AP1B134022550V5 | 0.371 | 0.003932 | 3.76 | -1.6456 | 1.92 | ANGPT1 |  |
| ASHG19LNC1A104431971V5 | 0.384 | 0.004819 | 3.63 | -1.8186 | 1.96 | SOX2-OT |  |
| ASHG19AP1B100012077V5 | 0.386 | 0.005071 | 3.6 | -1.8622 | 2.08 | FRK |  |
| ASHG19AP1B100078846V5 | 0.396 | 0.005721 | 3.53 | -1.9651 | 1.53 | ITGB1BP1 |  |
| ASHG19AP1B109582383V5 | 0.399 | 0.005987 | 3.5 | -2.0039 | 1.64 | TPD52 |  |
| ASHG19LNC1A100036657V5 | 0.405 | 0.006447 | 3.45 | -2.0672 | 1.78 | AC096708.3 | |
| ASHG19AP1B103391917V5 | 0.409 | 0.007075 | 3.4 | -2.1469 | 1.6 | ATP23 |  |
| ASHG19AP1B100227006V5 | 0.409 | 0.007176 | 3.39 | -2.159 | 1.52 | FANCF |  |
| ASHG19AP1B104193725V5 | 0.412 | 0.007591 | 3.36 | -2.2073 | 1.7 | MATN2 |  |
| ASHG19AP1B116773735V5 | 0.412 | 0.007659 | 3.35 | -2.2149 | 1.68 | RERG |  |
| ASHG19LNC1A100068455V5 | 0.412 | 0.007768 | 3.34 | -2.227 | 1.9 | AC078922.1 | |
| ASHG19LNC1A114391111V5 | 0.414 | 0.00796 | 3.33 | -2.248 | 1.53 | HERPUD1 |  |
| ASHG19LNC1A101339951V5 | 0.423 | 0.008361 | 3.3 | -2.2902 | 2 | DHRS7 |  |
| A_23_P11685 | 0.423 | 0.009747 | 3.21 | -2.4221 | 1.78 |  | A_23_P11685 |
| ASHG19LNC1A100015738V5 | 0.423 | 0.009804 | 3.2 | -2.4271 | 2.24 | AC009686.1 | |
| ASHG19LNC1A100064959V5 | 0.423 | 0.010181 | 3.18 | -2.4596 | 1.54 | AL159972.1 | |
| ASHGV40031044V5 | 0.423 | 0.010381 | 3.17 | -2.4762 | 1.54 | XLOC_l2_008924 | |
| ASHGV40051282V5 | 0.425 | 0.010543 | 3.16 | -2.4896 | 1.5 | XLOC_006934 | |
| ASHG19AP1B126968991V5 | 0.432 | 0.011552 | 3.1 | -2.5682 | 1.84 | ORMDL1 |  |
| ASHG19AP1B107729904V5 | 0.439 | 0.01309 | 3.03 | -2.676 | 1.52 | CREG1 |  |
| ASHGV40055173V5 | 0.439 | 0.014022 | 2.99 | -2.7352 | 1.8 | G089266 |  |
| ASHG19AP1B127181783V5 | 0.441 | 0.014547 | 2.97 | -2.7669 | 1.89 | MPZL2 |  |
| ASHG19LNC1A100097036V5 | 0.441 | 0.015024 | 2.95 | -2.7947 | 1.52 | AC023158.2 | |
| ASHGV40006307V5 | 0.441 | 0.015154 | 2.94 | -2.8022 | 2.93 | ATE1-AS1 |  |
| ASHG19LNC1A101814936V5 | 0.448 | 0.017554 | 2.86 | -2.9289 | 1.56 | ACAT2 |  |
| ASHG19LNC1A100048607V5 | 0.448 | 0.0181 | 2.84 | -2.9552 | 2.92 | AL731566.2 | |
| ASHG19AP1B130969784V5 | 0.448 | 0.019156 | 2.8 | -3.0041 | 1.59 | FZD6 |  |
| ASHGV40046165V5 | 0.448 | 0.02011 | 2.78 | -3.046 | 1.76 | G074631 |  |
| ASHG19LNC1A103690563V5 | 0.448 | 0.020125 | 2.77 | -3.0466 | 2.88 | CATG00000056335.1 | |
| ASHG19AP1B102803328V5 | 0.448 | 0.021079 | 2.75 | -3.0865 | 1.55 | RUNDC3B | |
| ASHG19AP1B111518950V5 | 0.448 | 0.021696 | 2.73 | -3.1114 | 1.87 | AP1S2 |  |
| ASHG19LNC1A100003872V5 | 0.448 | 0.022661 | 2.7 | -3.1488 | 1.58 | AL589765.6 | |
| ASHGV40020651V5 | 0.448 | 0.023279 | 2.69 | -3.172 | 1.99 | LOC653653 | |
| ASHG19LNC1A100047379V5 | 0.448 | 0.02333 | 2.69 | -3.1739 | 1.53 | AC104472.1 | |
| ASHGV40048701V5 | 0.455 | 0.025991 | 2.62 | -3.2668 | 1.59 | LINC01003 | |
| ASHG19AP1B122760875V5 | 0.455 | 0.026801 | 2.61 | -3.2931 | 1.54 | HPRT1 |  |
| ASHG19LNC1A100063740V5 | 0.455 | 0.026813 | 2.61 | -3.2935 | 1.67 | AL354977.1 | |
| ASHG19AP1B114335063V5 | 0.455 | 0.026818 | 2.61 | -3.2937 | 1.96 | RCN2 |  |
| ASHGV40012607V5 | 0.455 | 0.027119 | 2.6 | -3.3032 | 1.75 | G004281 |  |
| ASHG19AP1B100111522V5 | 0.455 | 0.027731 | 2.59 | -3.3224 | 1.8 | RAB3B |  |
| ASHGV40025440V5 | 0.459 | 0.03341 | 2.48 | -3.4822 | 1.59 | G039909 |  |
| ASHG19AP1B109960752V5 | 0.459 | 0.034589 | 2.46 | -3.5119 | 1.69 | DDX60 |  |
| ASHG19AP1B100015974V5 | 0.46 | 0.036186 | 2.43 | -3.5505 | 1.79 | CAMP |  |
| ASHG19LNC1A100059669V5 | 0.46 | 0.038365 | 2.39 | -3.6005 | 1.56 | AC009480.1 | |
| ASHGV40056123V5 | 0.461 | 0.039868 | 2.37 | -3.6333 | 1.88 | G025197 |  |
| ASHG19AP1B100080798V5 | 0.462 | 0.040535 | 2.36 | -3.6474 | 1.54 | DDIT4L |  |
| ASHG19AP1B127409724V5 | 0.462 | 0.0407 | 2.36 | -3.6509 | 1.72 | PAIP1 |  |
| ASHG19AP1B126723741V5 | 0.462 | 0.040878 | 2.36 | -3.6546 | 1.5 | PSMD10 |  |
| ASHG19AP1B110567155V5 | 0.462 | 0.041298 | 2.35 | -3.6634 | 1.9 | AGA |  |
| ASHG19AP1B100186238V5 | 0.462 | 0.041546 | 2.35 | -3.6684 | 2.07 | NPFFR1 |  |
| ASHG19LNC1ABL100000656V5 | 0.463 | 0.042004 | 2.34 | -3.6778 | 1.5 | FTX |  |
| ASHGV40055782V5 | 0.463 | 0.042917 | 2.33 | -3.6961 | 1.67 | G010573 |  |
| ASHG19LNC1A102917967V5 | 0.463 | 0.043028 | 2.33 | -3.6983 | 1.91 | HEXB |  |
| ASHG19AP1B119107268V5 | 0.463 | 0.043045 | 2.33 | -3.6986 | 1.62 | CCNG2 |  |
| ASHG19AP1B121209044V5 | 0.467 | 0.045116 | 2.3 | -3.7386 | 2.02 | CRISPLD1 |  |
| ASHG19AP1B122075685V5 | 0.467 | 0.047871 | 2.26 | -3.789 | 1.95 | TM9SF3 |  |
| ASHG19AP1B120075466V5 | 0.467 | 0.048377 | 2.26 | -3.7979 | 1.76 | CASD1 |  |
| ASHG19AP1B102120885V5 | 0.467 | 0.048457 | 2.26 | -3.7993 | 1.53 | SLC25A4 |  |
| ASHG19LNC1A111195442V5 | 0.185 | 0.000172 | -5.89 | 0.8768 | -1.68 | HAND2-AS1 | |
| ASHG19LNC1A110860318V5 | 0.185 | 0.000219 | -5.71 | 0.6957 | -1.78 | HAND2-AS1 | |
| ASHGV40012484V5 | 0.202 | 0.00025 | -5.61 | 0.5931 | -1.59 | G021657 |  |
| ASHG19LNC1A104008563V5 | 0.403 | 0.006253 | -3.47 | -2.0412 | -1.82 | SATB1-AS1 | |
| ASHGV40051066V5 | 0.421 | 0.008258 | -3.3 | -2.2796 | -1.87 | G082417 |  |
| ASHG19AP1B100033991V5 | 0.423 | 0.008903 | -3.26 | -2.3441 | -1.5 | STRA8 |  |
| ASHGV40007205V5 | 0.423 | 0.009143 | -3.24 | -2.3671 | -3.57 | AP002748.3 | |
| ASHG19AP1B119477949V5 | 0.423 | 0.009619 | -3.21 | -2.4107 | -1.61 | CCDC149 |  |
| ASHG19AP1B114648141V5 | 0.423 | 0.009903 | -3.2 | -2.4357 | -1.51 | UBC |  |
| ASHGV40027047V5 | 0.431 | 0.011192 | -3.12 | -2.541 | -1.59 | G044578 |  |
| ASHG19AP1B102019523V5 | 0.436 | 0.012236 | -3.07 | -2.6178 | -3.1 | SLC38A6 |  |
| ASHG19AP1B140337506V5 | 0.439 | 0.012578 | -3.05 | -2.6416 | -1.59 | PSMD8 |  |
| ASHG19LNC1A100080830V5 | 0.439 | 0.012673 | -3.05 | -2.648 | -1.68 | AL355075.4 | |
| ASHG19AP1B100249618V5 | 0.439 | 0.013418 | -3.01 | -2.6973 | -2.73 | CATG00000047891.1 | |
| ASHG19LNC1A114603925V5 | 0.439 | 0.014388 | -2.97 | -2.7574 | -1.52 | C3orf18 |  |
| ASHG19AP1B118361439V5 | 0.441 | 0.014553 | -2.97 | -2.7673 | -1.94 | DAZAP2 |  |
| ASHGV40026633V5 | 0.445 | 0.015693 | -2.92 | -2.8323 | -2.01 | G043166 |  |
| ASHGV40044376V5 | 0.447 | 0.016652 | -2.89 | -2.8834 | -2.02 | PDE10A |  |
| ASHG19LNC1A100100719V5 | 0.448 | 0.017494 | -2.86 | -2.9259 | -2.51 | ACP1 |  |
| ASHG19AP1B143318076V5 | 0.448 | 0.017591 | -2.85 | -2.9307 | -1.86 | RPL17 |  |
| ASHGV40001061V5 | 0.448 | 0.018606 | -2.82 | -2.979 | -1.99 | AC006482.1 | |
| ASHG19LNC1A109193468V5 | 0.448 | 0.018743 | -2.82 | -2.9853 | -1.87 | LINC00893 | |
| ASHG19LNC1A109848777V5 | 0.448 | 0.019713 | -2.79 | -3.0288 | -1.71 | AC011462.1 | |
| ASHGV40046878V5 | 0.448 | 0.019811 | -2.78 | -3.0331 | -2.18 | G077303 |  |
| ASHGV40004143V5 | 0.448 | 0.019917 | -2.78 | -3.0377 | -3.24 | OGFR-AS1 | |
| ASHG19AP1B129558042V5 | 0.448 | 0.020257 | -2.77 | -3.0523 | -1.52 | ALAD |  |
| ASHG19AP1B107320883V5 | 0.448 | 0.021515 | -2.74 | -3.1042 | -2.66 | 45172 |  |
| ASHGV40051636V5 | 0.448 | 0.021665 | -2.73 | -3.1101 | -2.36 | G084241 |  |
| ASHGV40009963V5 | 0.455 | 0.028058 | -2.58 | -3.3325 | -1.5 | G018241 |  |
| ASHG19LNC1A100044409V5 | 0.455 | 0.028321 | -2.57 | -3.3405 | -1.9 | AC005479.1 | |
| ASHG19AP1B142140940V5 | 0.455 | 0.028649 | -2.57 | -3.3504 | -1.54 | ECM1 |  |
| ASHG19AP1B102725107V5 | 0.455 | 0.028752 | -2.56 | -3.3535 | -2.28 | SLC4A11 |  |
| ASHG19AP1B118562524V5 | 0.455 | 0.031176 | -2.52 | -3.4229 | -2.26 | CATG00000116938.1 | |
| ASHG19AP1B134457611V5 | 0.455 | 0.031247 | -2.52 | -3.4249 | -1.57 | S100A13 |  |
| ASHG19AP1B115421193V5 | 0.457 | 0.032439 | -2.49 | -3.457 | -2.29 | POMC |  |
| ASHG19LNC1A104387517V5 | 0.459 | 0.033389 | -2.48 | -3.4817 | -1.91 | VPS52 |  |
| ASHGV40039721V5 | 0.459 | 0.033948 | -2.47 | -3.4959 | -1.58 | XLOC_003804 | |
| ASHG19AP1B131783095V5 | 0.459 | 0.034885 | -2.45 | -3.5192 | -1.57 | ATG5 |  |
| ASHG19AP1B104754252V5 | 0.459 | 0.034941 | -2.45 | -3.5206 | -3.25 | OR6N1 |  |
| ASHG19AP1B100126785V5 | 0.459 | 0.034992 | -2.45 | -3.5218 | -1.55 | EGR1 |  |
| ASHGV40003805V5 | 0.459 | 0.035077 | -2.45 | -3.5239 | -1.5 | ACBD6 |  |
| ASHG19AP1B117546460V5 | 0.459 | 0.03509 | -2.45 | -3.5242 | -2.08 | MYH7B |  |
| ASHG19AP1B134254955V5 | 0.46 | 0.035189 | -2.45 | -3.5266 | -1.5 | ITK |  |
| ASHG19LNC1A106054216V5 | 0.46 | 0.035518 | -2.44 | -3.5346 | -1.94 | BX890604.2 | |
| ASHG19AP1B114968547V5 | 0.46 | 0.035584 | -2.44 | -3.5362 | -1.89 | HTR3E |  |
| ASHGV40030427V5 | 0.46 | 0.035623 | -2.44 | -3.5371 | -1.66 | G049000 |  |
| ASHG19AP1B140032216V5 | 0.46 | 0.035634 | -2.44 | -3.5374 | -1.6 | PCSK4 |  |
| ASHG19AP1B102558364V5 | 0.46 | 0.036237 | -2.43 | -3.5517 | -1.76 | RPL17 |  |
| ASHG19LNC1A107227596V5 | 0.46 | 0.037822 | -2.4 | -3.5883 | -2.53 | AC004943.2 | |
| ASHG19LNC1A102422395V5 | 0.46 | 0.038216 | -2.4 | -3.5972 | -2.25 | SATB1-AS1 | |
| ASHG19LNC1A103973269V5 | 0.461 | 0.039843 | -2.37 | -3.6328 | -3.15 | OR6N1 |  |
| ASHG19AP1B125589482V5 | 0.461 | 0.039911 | -2.37 | -3.6342 | -1.53 | GSDME |  |
| ASHG19LNC1A100096441V5 | 0.461 | 0.03996 | -2.37 | -3.6353 | -2.56 | AC083906.3 | |
| ASHG19LNC1A104430292V5 | 0.461 | 0.040031 | -2.37 | -3.6368 | -2.2 | AP002026.1 | |
| ASHG19AP1B119409788V5 | 0.462 | 0.040514 | -2.36 | -3.647 | -2.68 | SCNN1G |  |
| ASHG19AP1B133861561V5 | 0.462 | 0.040807 | -2.36 | -3.6531 | -2.37 | MED15 |  |
| ASHG19LNC1A112729097V5 | 0.462 | 0.040889 | -2.36 | -3.6549 | -1.98 | AC090152.1 | |
| ASHGV40001246V5 | 0.464 | 0.043653 | -2.32 | -3.7106 | -1.77 | AL137847.2 | |
| ASHGV40033567V5 | 0.467 | 0.045708 | -2.29 | -3.7497 | -3.28 | AL020994.3 | |
| ASHG19LNC1A100097050V5 | 0.467 | 0.048195 | -2.26 | -3.7947 | -1.62 | AC037198.1 | |

Sheet 2

| Sheet 2  **Significantly differentially gene exoression in GSE205398** | | | | | |  |  |  |  |  |
| --- | --- | --- | --- | --- | --- | --- | --- | --- | --- | --- |
| **GeneID** | **padj** | **pvalue** | **lfcSE** | **stat** | **log2FoldChange** | **baseMean** | **Symbol** | **Description** | **Synonyms** | **GeneType** |
| 1469 | 1.54E-05 | 7.27E-10 | 0.355 | 6.16007 | 2.185666 | 94.84 | CACNA1H | cystatin SN | | protein-coding |
| 1.05E+08 | 0.000108 | 1.02E-08 | 0.995 | 5.727509 | 5.700658 | 25.42 | LOC105377177 | uncharacterized LOC105377177 | | ncRNA |
| 1472 | 0.008307 | 1.18E-06 | 0.768 | 4.859604 | 3.734205 | 28.62 | CST4 | KCNQ1 |  | protein-coding |
| 126006 | 0.011358 | 3.75E-06 | 0.402 | 4.624637 | 1.857921 | 53.95 | PCP2 | Purkinje cell protein 2 | GPSM4\|PCD5 | protein-coding |
| 174 | 0.037676 | 2.13E-05 | 1.508 | 4.250401 | 6.410658 | 1153.9 | AFP | alpha fetoprotein | AFPD\|FETA\|HPAFP | protein-coding |
| 727764 | 0.049056 | 3.24E-05 | 0.506 | 4.155797 | 2.104101 | 666.48 | MAFIP | MAFF interacting protein (pseudogene) | MIP\|TEKT4P4\|pp5644 | pseudo |
| 6323 | 0.057641 | 4.41E-05 | 0.867 | 4.085126 | 3.541697 | 18.12 | SCN1A | sodium voltage-gated channel alpha subunit 1 | DEE6\|DEE6A\|DEE6B\|DRVT\|EIEE6\|FEB3\|FEB3A\|FHM3\|GEFSP2\|HBSCI\|NAC1\|Nav1.1\|SCN1\|SMEI | protein-coding |
| 115749 | 0.057641 | 4.45E-05 | 0.855 | 4.082788 | 3.490977 | 15.35 | C12orf56 | chromosome 12 open reading frame 56 | | protein-coding |
| 4069 | 0.061914 | 5.82E-05 | 0.459 | 4.020038 | 1.846492 | 249.2 | LYZ | lysozyme | LYZF1\|LZM | protein-coding |
| 213 | 0.107514 | 1.17E-04 | 1.471 | 3.852927 | 5.669101 | 334.05 | ALB | albumin | FDAHT\|HSA\|PRO0883\|PRO0903\|PRO1341 | protein-coding |
| 2243 | 0.110732 | 1.30E-04 | 0.993 | 3.826825 | 3.800842 | 50.19 | FGA | fibrinogen alpha chain | Fib2 | protein-coding |
| 163404 | 0.125095 | 1.65E-04 | 0.681 | 3.766848 | 2.564345 | 21.69 | PLPPR5 | phospholipid phosphatase related 5 | LPPR5\|PAP2\|PAP2D\|PRG5 | protein-coding |
| 1.05E+08 | 0.16605 | 2.51E-04 | 0.609 | 3.661447 | 2.229939 | 38 | LOC105377979 | uncharacterized LOC105377979 | | ncRNA |
| 1.1E+08 | 0.182748 | 3.36E-04 | 0.436 | 3.585531 | 1.56279 | 1269.32 | RNA28SN4 | RNA, 28S ribosomal N4 | RN28S1\|RNA28S5 | rRNA |
| 5629 | 0.193536 | 3.75E-04 | 0.614 | 3.557416 | 2.184766 | 137.59 | PROX1 | prospero homeobox 1 | | protein-coding |
| 343450 | 0.215767 | 4.52E-04 | 0.748 | 3.507648 | 2.622447 | 21.55 | KCNT2 | potassium sodium-activated channel subfamily T member 2 | DEE57\|EIEE57\|KCa4.2\|SLICK\|SLO2.1 | protein-coding |
| 3698 | 0.260867 | 6.16E-04 | 0.704 | 3.424634 | 2.410508 | 84.59 | ITIH2 | inter-alpha-trypsin inhibitor heavy chain 2 | H2P\|SHAP | protein-coding |
| 23316 | 0.260867 | 6.14E-04 | 0.563 | 3.425389 | 1.928836 | 18.7 | CUX2 | cut like homeobox 2 | CDP2\|CUTL2\|DEE67\|EIEE67 | protein-coding |
| 1.07E+08 | 0.262743 | 6.32E-04 | 0.458 | 3.417299 | 1.566747 | 339.1 | RNA28SN1 | RNA, 28S ribosomal N1 | RNA28S4 | rRNA |
| 439936 | 0.266877 | 6.68E-04 | 0.646 | 3.40255 | 2.197312 | 20.83 | LINC02899 | long intergenic non-protein coding RNA 2899 | C5orf17 | ncRNA |
| 140828 | 0.357951 | 9.63E-04 | 0.629 | 3.301117 | 2.075705 | 63.45 | LINC00261 | long intergenic non-protein coding RNA 261 | ALIEN\|C20orf56\|DEANR1\|FALCOR\|HCCDR1\|LCAL62\|NCRNA00261\|TCONS_00027846\|onco-lncRNA-17 | ncRNA |
| 1E+08 | 0.421181 | 1.33E-03 | 0.718 | 3.209014 | 2.303697 | 121.61 | TEKT4P2 | tektin 4 pseudogene 2 | MAFIPL\|TEKT4P | pseudo |
| 1E+08 | 0.436283 | 1.46E-03 | 0.486 | 3.182116 | 1.545961 | 710.88 | RNA28SN5 | RNA, 28S ribosomal N5 | RN28S1\|RNA28S5 | rRNA |
| 338 | 0.44608 | 1.56E-03 | 1.12 | 3.163652 | 3.542117 | 1134.1 | APOB | apolipoprotein B | FCHL2\|FLDB\|LDLCQ4\|apoB-100\|apoB-48 | protein-coding |
| 1645 | 0.451405 | 1.60E-03 | 0.548 | 3.156284 | 1.7296 | 65.29 | AKR1C1 | aldo-keto reductase family 1 member C1 | 2-ALPHA-HSD\|20-ALPHA-HSD\|C9\|DD1\|DD1/DD2\|DDH\|DDH1\|H-37\|HAKRC\|HBAB\|MBAB | protein-coding |
| 431705 | 0.519004 | 1.96E-03 | 0.533 | 3.096843 | 1.650026 | 42.59 | ASTL | astacin like metalloendopeptidase | OOMD11\|SAS1B | protein-coding |
| 158248 | 0.519004 | 1.98E-03 | 0.795 | 3.092586 | 2.458294 | 9.99 | TTC16 | tetratricopeptide repeat domain 16 | | protein-coding |
| 6398 | 0.519004 | 1.97E-03 | 0.522 | 3.094952 | 1.616781 | 89.59 | SECTM1 | secreted and transmembrane 1 | K12\|SECTM | protein-coding |
| 26659 | 0.519004 | 1.93E-03 | 0.736 | 3.100516 | 2.281073 | 21.28 | OR7A5 | olfactory receptor family 7 subfamily A member 5 | HTPCR2 | protein-coding |
| 1.05E+08 | 0.559307 | 2.17E-03 | 0.673 | 3.06564 | 2.062643 | 40.78 | LOC105374029 | uncharacterized LOC105374029 | | ncRNA |
| 50614 | 0.559307 | 2.24E-03 | 0.605 | 3.055897 | 1.848943 | 17.64 | GALNT9 | polypeptide N-acetylgalactosaminyltransferase 9 | GALNAC-T9\|GALNACT9 | protein-coding |
| 1.1E+08 | 0.602908 | 2.57E-03 | 0.516 | 3.0152 | 1.557109 | 164.96 | RNA18SN2 | RNA, 18S ribosomal N2 | | rRNA |
| 204962 | 0.603105 | 2.65E-03 | 0.665 | 3.005974 | 1.99811 | 19.54 | SLC44A5 | solute carrier family 44 member 5 | CTL5 | protein-coding |
| 1.02E+08 | 0.612855 | 2.79E-03 | 0.733 | 2.990009 | 2.190652 | 13.17 | TROAP-AS1 | TROAP and PRPH antisense RNA 1 | | ncRNA |
| 5053 | 0.612855 | 2.75E-03 | 0.846 | 2.99394 | 2.533646 | 95.81 | PAH | phenylalanine hydroxylase | PH\|PKU\|PKU1 | protein-coding |
| 1.07E+08 | 0.612855 | 2.73E-03 | 0.58 | 2.99649 | 1.738712 | 607.03 | RNA18SN1 | RNA, 18S ribosomal N1 | RNA18S4 | rRNA |
| 6364 | 0.630245 | 2.94E-03 | 0.879 | 2.973429 | 2.613827 | 15.71 | CCL20 | C-C motif chemokine ligand 20 | CKb4\|Exodus\|LARC\|MIP-3-alpha\|MIP-3a\|MIP3A\|SCYA20\|ST38 | protein-coding |
| 7018 | 0.701809 | 3.54E-03 | 1.093 | 2.916104 | 3.186032 | 153.54 | TF | transferrin | HEL-S-71p\|PRO1557\|PRO2086\|TFQTL1 | protein-coding |
| 29974 | 0.726204 | 3.99E-03 | 1.716 | 2.879266 | 4.940867 | 100.52 | A1CF | APOBEC1 complementation factor | ACF\|ACF64\|ACF65\|APOBEC1CF\|ASP | protein-coding |
| 1.05E+08 | 0.726204 | 3.83E-03 | 0.558 | 2.891748 | 1.613649 | 23.72 | LOC105370091 | uncharacterized LOC105370091 | | ncRNA |
| 1.05E+08 | 0.762133 | 4.35E-03 | 0.592 | 2.85141 | 1.687529 | 115.74 | LOC105379271 | uncharacterized LOC105379271 | | ncRNA |
| 51557 | 0.782584 | 4.62E-03 | 0.61 | 2.832599 | 1.727588 | 58.28 | LGSN | lengsin, lens protein with glutamine synthetase domain | GLULD1\|LGS | protein-coding |
| 1.05E+08 | 0.826414 | 5.19E-03 | 0.581 | 2.795138 | 1.623123 | 26.25 | LOC105370821 | uncharacterized LOC105370821 | | ncRNA |
| 2153 | 0.885031 | 6.06E-03 | 0.634 | 2.74468 | 1.739483 | 37.67 | F5 | coagulation factor V | FVL\|PCCF\|RPRGL1\|THPH2 | protein-coding |
| 57497 | 0.885031 | 6.03E-03 | 1.052 | 2.745969 | 2.889936 | 18.68 | LRFN2 | leucine rich repeat and fibronectin type III domain containing 2 | FIGLER2\|KIAA1246\|SALM1 | protein-coding |
| 23089 | 0.885031 | 5.90E-03 | 0.924 | 2.753047 | 2.543079 | 451.45 | PEG10 | paternally expressed 10 | EDR\|HB-1\|MEF3L\|Mar2\|Mart2\|RGAG3\|RTL2\|SIRH1 | protein-coding |
| 1.08E+08 | 0.885031 | 5.75E-03 | 0.576 | 2.761531 | 1.589543 | 38.66 | LOC107984007 | uncharacterized LOC107984007 | | ncRNA |
| 53828 | 0.885031 | 5.95E-03 | 0.567 | 2.75038 | 1.560457 | 26.14 | FXYD4 | FXYD domain containing ion transport regulator 4 | CHIF | protein-coding |
| 1.05E+08 | 0.903446 | 6.35E-03 | 0.558 | 2.728953 | 1.523098 | 18.4 | LOC105371425 | uncharacterized LOC105371425 | | ncRNA |
| 54102 | 0.91238 | 6.62E-03 | 0.766 | 2.715573 | 2.081191 | 14.87 | CLIC6 | chloride intracellular channel 6 | CLIC1L | protein-coding |
| 26 | 0.917266 | 7.86E-03 | 0.956 | 2.657966 | 2.541323 | 53.94 | AOC1 | amine oxidase copper containing 1 | ABP\|ABP1\|DAO\|DAO1\|KAO | protein-coding |
| 1.02E+08 | 0.917266 | 7.21E-03 | 0.669 | 2.686861 | 1.797376 | 56.3 | DISC1FP1 | DISC1 fusion partner 1 | Boymaw | ncRNA |
| 1.01E+08 | 0.917266 | 7.82E-03 | 0.714 | 2.659825 | 1.899602 | 15.87 | CTXND1 | cortexin domain containing 1 | LINC01314 | protein-coding |
| 1.1E+08 | 0.917266 | 6.86E-03 | 0.585 | 2.70351 | 1.582658 | 223.55 | RNA18SN3 | RNA, 18S ribosomal N3 | | rRNA |
| 1.05E+08 | 0.931265 | 8.18E-03 | 1.001 | 2.644733 | 2.646422 | 17.72 | LOC105370196 | uncharacterized LOC105370196 | | ncRNA |
| 3127 | 0.951524 | 8.40E-03 | 0.592 | 2.635623 | 1.559207 | 36.27 | HLA-DRB5 | major histocompatibility complex, class II, DR beta 5 | HLA-DRB5* | protein-coding |
| 1.02E+08 | 0.999946 | 4.19E-02 | 0.742 | 2.034538 | 1.509404 | 9.89 | LINC01756 | long intergenic non-protein coding RNA 1756 | | ncRNA |
| 6280 | 0.999946 | 1.55E-02 | 0.71 | 2.420618 | 1.717692 | 42.87 | S100A9 | S100 calcium binding protein A9 | 60B8AG\|CAGB\|CFAG\|CGLB\|L1AG\|LIAG\|MAC387\|MIF\|MRP14\|NIF\|P14\|S100-A9 | protein-coding |
| 1.02E+08 | 0.999946 | 2.45E-02 | 1.094 | 2.248615 | 2.458864 | 32.44 | LINC01293 | long intergenic non-protein coding RNA 1293 | | ncRNA |
| 1.05E+08 | 0.999946 | 1.56E-02 | 0.646 | 2.418452 | 1.561773 | 32.44 | LOC105373611 | uncharacterized LOC105373611 | | ncRNA |
| 2888 | 0.999946 | 3.03E-02 | 0.94 | 2.166651 | 2.036515 | 12.92 | GRB14 | growth factor receptor bound protein 14 | | protein-coding |
| 6335 | 0.999946 | 9.57E-03 | 0.761 | 2.590833 | 1.972695 | 45.16 | SCN9A | sodium voltage-gated channel alpha subunit 9 | ETHA\|FEB3B\|GEFSP7\|HSAN2D\|NE-NA\|NENA\|Nav1.7\|PN1\|SFNP | protein-coding |
| 1.08E+08 | 0.999946 | 3.34E-02 | 1.116 | 2.127267 | 2.37376 | 13.83 | LOC107985962 | uncharacterized LOC107985962 | | ncRNA |
| 1E+08 | 0.999946 | 1.35E-02 | 0.62 | 2.470261 | 1.531896 | 17.25 | FGD5P1 | FYVE, RhoGEF and PH domain containing 5 pseudogene 1 | | pseudo |
| 2891 | 0.999946 | 2.55E-02 | 1.033 | 2.233885 | 2.307001 | 40.45 | GRIA2 | glutamate ionotropic receptor AMPA type subunit 2 | GLUR2\|GLURB\|GluA2\|GluR-K2\|HBGR2\|NEDLIB\|gluR-2\|gluR-B | protein-coding |
| 1.05E+08 | 0.999946 | 1.89E-02 | 0.668 | 2.347168 | 1.567446 | 14.43 | LOC105379169 | uncharacterized LOC105379169 | | ncRNA |
| 57451 | 0.999946 | 4.73E-02 | 0.8 | 1.983382 | 1.586066 | 34.77 | TENM2 | teneurin transmembrane protein 2 | ODZ2\|TEN-M2\|TEN2\|TNM2\|ten-2 | protein-coding |
| 1.05E+08 | 0.999946 | 3.24E-02 | 0.858 | 2.138919 | 1.836113 | 11.34 | LOC105374995 | |  |  |
| 389376 | 0.999946 | 2.95E-02 | 0.754 | 2.176649 | 1.641982 | 22.05 | SFTA2 | surfactant associated 2 | GSGL541\|SFTPG\|SP-G\|UNQ541 | protein-coding |
| 401331 | 0.999946 | 1.86E-02 | 0.64 | 2.353543 | 1.506663 | 15.94 | RASA4CP | RAS p21 protein activator 4C, pseudogene | RASA4P | pseudo |
| 5649 | 0.999946 | 1.78E-02 | 0.819 | 2.37057 | 1.94253 | 73.68 | RELN | reelin | ETL7\|LIS2\|PRO1598\|RL | protein-coding |
| 1.01E+08 | 0.999946 | 2.59E-02 | 0.698 | 2.227643 | 1.555358 | 53.05 | COMETT | cytosolic oncogenic antisense to MET transcript | COMET\|LINC01510 | ncRNA |
| 1.05E+08 | 0.999946 | 2.73E-02 | 0.738 | 2.206877 | 1.628631 | 31.83 | LOC105375523 | uncharacterized LOC105375523 | | ncRNA |
| 760 | 0.999946 | 1.39E-02 | 0.693 | 2.459893 | 1.705156 | 42.38 | CA2 | carbonic anhydrase 2 | CA-II\|CAC\|CAII\|Car2\|HEL-76\|HEL-S-282 | protein-coding |
| 259 | 0.999946 | 4.65E-02 | 0.997 | 1.990746 | 1.984816 | 37.68 | AMBP | alpha-1-microglobulin/bikunin precursor | A1M\|EDC1\|HCP\|HI30\|IATIL\|ITI\|ITIL\|ITILC\|UTI | protein-coding |
| 118856 | 0.999946 | 3.45E-02 | 0.92 | 2.113663 | 1.94547 | 15.64 | MMP21 | matrix metallopeptidase 21 | HTX7\|MMP-21 | protein-coding |
| 85388 | 0.999946 | 1.13E-02 | 0.658 | 2.533773 | 1.666488 | 14.53 | SNORD14B | small nucleolar RNA, C/D box 14B | RNU14B\|U14\|U14B | snoRNA |
| 1.05E+08 | 0.999946 | 2.34E-02 | 0.906 | 2.266127 | 2.052693 | 35.95 | CSRP3-AS1 | CSRP3 and E2F8 antisense RNA 1 | | ncRNA |
| 8091 | 0.999946 | 1.91E-02 | 1.223 | 2.343352 | 2.866285 | 148.06 | HMGA2 | high mobility group AT-hook 2 | BABL\|HMGI-C\|HMGIC\|LIPO\|SRS5\|STQTL9 | protein-coding |
| 27345 | 0.999946 | 1.22E-02 | 0.607 | 2.505793 | 1.521211 | 87.74 | KCNMB4 | potassium calcium-activated channel subfamily M regulatory beta subunit 4 | | protein-coding |
| 144321 | 0.999946 | 1.69E-02 | 0.795 | 2.388026 | 1.898372 | 13.38 | GLIPR1L2 | GLIPR1 like 2 | | protein-coding |
| 1.01E+08 | 0.999946 | 3.54E-02 | 0.788 | 2.103989 | 1.65817 | 10.1 | LINC02361 | long intergenic non-protein coding RNA 2361 | | ncRNA |
| 1.08E+08 | 0.999946 | 3.38E-02 | 0.994 | 2.122778 | 2.108999 | 11.93 | LOC107984695 | |  |  |
| 123041 | 0.999946 | 4.63E-02 | 0.881 | 1.992555 | 1.755373 | 58.59 | SLC24A4 | solute carrier family 24 member 4 | AI2A5\|NCKX4\|SHEP6\|SLC24A2 | protein-coding |
| 1E+08 | 0.999946 | 2.77E-02 | 0.767 | 2.201252 | 1.688986 | 16.61 | TRR-CCT3-1 | tRNA-Arg (anticodon CCT) 3-1 | TRNAR18 | tRNA |
| 1E+08 | 0.999946 | 3.05E-02 | 0.699 | 2.163257 | 1.512236 | 10.54 | MIR320C1 | microRNA 320c-1 | MIR320C-1\|MIRN320C1\|hsa-mir-320c-1\|mir-320c-1 | ncRNA |
| 342979 | 0.999946 | 2.01E-02 | 0.685 | 2.324462 | 1.593253 | 38.49 | PALM3 | paralemmin 3 | | protein-coding |
| 3755 | 0.999946 | 1.28E-02 | 0.907 | 2.489995 | 2.258169 | 62.39 | KCNG1 | potassium voltage-gated channel modifier subfamily G member 1 | K13\|KCNG\|KV6.1\|kH2 | protein-coding |
| 3772 | 0.999946 | 4.72E-02 | 1.052 | 1.984857 | 2.087116 | 18.74 | KCNJ15 | potassium inwardly rectifying channel subfamily J member 15 | IRKK\|KIR1.3\|KIR4.2 | protein-coding |
| 84700 | 0.999946 | 1.40E-02 | 0.775 | 2.456485 | 1.9037 | 24.95 | MYO18B | myosin XVIIIB | KFS4 | protein-coding |
| 1E+08 | 0.999946 | 4.32E-02 | 0.81 | 2.021329 | 1.637264 | 724.25 | RNA18SN5 | RNA, 18S ribosomal N5 | RN18S1\|RNA18S5 | rRNA |
| 1E+08 | 0.999946 | 4.16E-02 | 0.92 | 2.037713 | 1.87391 | 76.72 | LOC100233156 | tektin 4 pseudogene | | pseudo |
| 115572 | NA | 3.77E-02 | 0.808 | 2.077901 | 1.678531 | 7.82 | TENT5B | terminal nucleotidyltransferase 5B | FAM46\|FAM46B | protein-coding |
| 1.03E+08 | NA | 1.29E-02 | 0.971 | 2.48589 | 2.413753 | 9.6 | LOC102724096 | uncharacterized LOC102724096 | | ncRNA |
| 387264 | NA | 2.92E-02 | 0.761 | 2.18076 | 1.659527 | 9.52 | KRTAP5-1 | keratin associated protein 5-1 | KRN1L\|KRTAP5.1 | protein-coding |
| 1.02E+08 | NA | 6.05E-03 | 0.889 | 2.745278 | 2.440393 | 8.13 | TMEM123-DT | TMEM123 divergent transcript | | ncRNA |
| 1.05E+08 | NA | 2.13E-02 | 0.726 | 2.302193 | 1.672175 | 9.51 | LOC105370033 | |  |  |
| 1.08E+08 | NA | 7.41E-04 | 1.143 | 3.373807 | 3.856945 | 7.58 | LOC107984456 | |  |  |
| 388323 | NA | 3.66E-02 | 0.816 | 2.09041 | 1.706034 | 8.04 | GLTPD2 | glycolipid transfer protein domain containing 2 | | protein-coding |
| 113691 | NA | 3.68E-02 | 0.769 | 2.087871 | 1.605348 | 9.19 | TUBA3FP | tubulin alpha 3f pseudogene | | pseudo |
| 3001 | 0.010924 | 3.09E-06 | 0.328 | -4.6645 | -1.53109 | 203.58 | GZMA | granzyme A | CTLA3\|HFSP | protein-coding |
| 387590 | 0.167221 | 0.00028 | 0.624 | -3.63311 | -2.26714 | 36.19 | TPTEP1 | TPTE pseudogene 1 | psiTPTE22 | pseudo |
| 1.05E+08 | 0.208177 | 0.000423 | 0.472 | -3.52561 | -1.66474 | 29.71 | LOC105373856 | uncharacterized LOC105373856 | | ncRNA |
| 1.08E+08 | 0.260867 | 0.000589 | 0.584 | -3.43672 | -2.00744 | 21.48 | LOC107984806 | uncharacterized LOC107984806 | | ncRNA |
| 22885 | 0.383695 | 0.00113 | 0.588 | -3.25673 | -1.91549 | 324.5 | ABLIM3 | actin binding LIM protein family member 3 | HMFN1661 | protein-coding |
| 54715 | 0.383695 | 0.00114 | 0.693 | -3.25325 | -2.25421 | 26.47 | RBFOX1 | RNA binding fox-1 homolog 1 | 2BP1\|A2BP1\|FOX-1\|FOX1\|HRNBP1 | protein-coding |
| 9407 | 0.421181 | 0.00132 | 0.488 | -3.2113 | -1.56861 | 45.62 | TMPRSS11D | transmembrane serine protease 11D | ASP\|HAT | protein-coding |
| 1.05E+08 | 0.559307 | 0.00221 | 0.675 | -3.06057 | -2.06445 | 20.63 | LOC105369656 | uncharacterized LOC105369656 | | ncRNA |
| 9153 | 0.581939 | 0.00241 | 0.938 | -3.03469 | -2.84634 | 51.46 | SLC28A2 | solute carrier family 28 member 2 | CNT2\|HCNT2\|HsT17153\|SPNT1 | protein-coding |
| 55799 | 0.602908 | 0.0026 | 0.53 | -3.01178 | -1.59648 | 41.18 | CACNA2D3 | calcium voltage-gated channel auxiliary subunit alpha2delta 3 | HSA272268 | protein-coding |
| 10891 | 0.636151 | 0.00309 | 0.582 | -2.95838 | -1.72097 | 253.05 | PPARGC1A | PPARG coactivator 1 alpha | LEM6\|PGC-1(alpha)\|PGC-1alpha\|PGC-1v\|PGC1\|PGC1A\|PPARGC1 | protein-coding |
| 23783 | 0.66723 | 0.00331 | 0.795 | -2.93769 | -2.33692 | 13.9 | ANKRD62P1-PARP4P3 | ANKRD62P1-PARP4P3 readthrough, transcribed pseudogene | VWFP1-ANKRD62P1-PARP4P3 | pseudo |
| 3002 | 0.717972 | 0.00369 | 0.537 | -2.9032 | -1.56046 | 83.54 | GZMB | granzyme B | C11\|CCPI\|CGL-1\|CGL1\|CSP-B\|CSPB\|CTLA1\|CTSGL1\|HLP\|SECT | protein-coding |
| 650226 | 0.726204 | 0.00404 | 0.743 | -2.87467 | -2.13542 | 32.09 | LOC650226 | ankyrin repeat domain containing 26 pseudogene | | pseudo |
| 2679 | 0.7646 | 0.0044 | 0.855 | -2.84776 | -2.43432 | 43.34 | GGT3P | gamma-glutamyltransferase 3 pseudogene | GGT3 | pseudo |
| 1.02E+08 | 0.826414 | 0.00518 | 0.733 | -2.79579 | -2.04847 | 15.28 | LOC101928540 | uncharacterized LOC101928540 | | ncRNA |
| 339967 | 0.917266 | 0.00752 | 0.754 | -2.67296 | -2.01637 | 14.09 | TMPRSS11A | transmembrane serine protease 11A | ECRG1\|HATL1\|HESP | protein-coding |
| 1E+08 | 0.917266 | 0.00757 | 0.563 | -2.67071 | -1.50433 | 19.67 | SNORD58C | small nucleolar RNA, C/D box 58C | U58 | snoRNA |
| 148198 | 0.917266 | 0.00711 | 0.782 | -2.69173 | -2.10556 | 11.4 | ZNF98 | zinc finger protein 98 | F7175\|ZNF739 | protein-coding |
| 200879 | 0.931265 | 0.00814 | 0.822 | -2.6462 | -2.1763 | 27.61 | LIPH | lipase H | AH\|ARWH2\|HYPT7\|LAH2\|LPDLR\|PLA1B\|mPA-PLA1 | protein-coding |
| 1.05E+08 | 0.954644 | 0.00865 | 0.577 | -2.62554 | -1.51519 | 20.99 | LOC105374260 | uncharacterized LOC105374260 | | ncRNA |
| 1.02E+08 | 0.958595 | 0.00881 | 0.724 | -2.6195 | -1.8976 | 13.23 | ALG14-AS1 | ALG14 antisense RNA 1 | | ncRNA |
| 3809 | 0.958595 | 0.00876 | 0.573 | -2.62132 | -1.50091 | 33.23 | KIR2DS4 | killer cell immunoglobulin like receptor, two Ig domains and short cytoplasmic tail 4 | CD158I\|KIR-2DS4\|KIR1D\|KIR412\|KKA3\|NKAT-8\|NKAT8 | protein-coding |
| 1.01E+08 | 0.999946 | 0.0313 | 0.944 | -2.15282 | -2.03153 | 10.92 | LINC02606 | long intergenic non-protein coding RNA 2606 | | ncRNA |
| 1.02E+08 | 0.999946 | 0.0145 | 0.683 | -2.44496 | -1.66993 | 66.92 | LOC101928216 | |  |  |
| 1.05E+08 | 0.999946 | 0.0265 | 0.896 | -2.21948 | -1.98856 | 17.3 | LOC105371651 | uncharacterized LOC105371651 | | ncRNA |
| 1.08E+08 | 0.999946 | 0.0487 | 0.809 | -1.97138 | -1.59534 | 12.68 | LOC107985908 | uncharacterized LOC107985908 | | ncRNA |
| 1.02E+08 | 0.999946 | 0.0461 | 0.762 | -1.99433 | -1.51953 | 30.63 | LINC01266 | long intergenic non-protein coding RNA 1266 | | ncRNA |
| 1.05E+08 | 0.999946 | 0.025 | 1.113 | -2.241 | -2.49464 | 19.75 | LOC105377567 | uncharacterized LOC105377567 | | ncRNA |
| 1.05E+08 | 0.999946 | 0.0218 | 0.679 | -2.29299 | -1.55785 | 15.76 | LOC105374649 | uncharacterized LOC105374649 | | ncRNA |
| 1.02E+08 | 0.999946 | 0.0427 | 0.86 | -2.02649 | -1.74225 | 16.56 | LOC101928004 | uncharacterized LOC101928004 | | ncRNA |
| 1.08E+08 | 0.999946 | 0.0477 | 1.354 | -1.97996 | -2.6801 | 18.34 | LOC107987166 | uncharacterized LOC107987166 | | ncRNA |
| 1.08E+08 | 0.999946 | 0.0214 | 0.763 | -2.30141 | -1.75668 | 10.22 | LOC107984548 | uncharacterized LOC107984548 | | ncRNA |
| 144423 | 0.999946 | 0.0311 | 0.72 | -2.15523 | -1.55145 | 154.93 | GLT1D1 | glycosyltransferase 1 domain containing 1 | | protein-coding |
| 28521 | 0.999946 | 0.0216 | 0.717 | -2.29807 | -1.64819 | 10.2 | TRDJ2 | T cell receptor delta joining 2 | | other |
| 1.05E+08 | 0.999946 | 0.0228 | 0.749 | -2.27727 | -1.70582 | 14.17 | LOC105370861 | uncharacterized LOC105370861 | | ncRNA |
| 1.05E+08 | 0.999946 | 0.0191 | 0.777 | -2.34354 | -1.81983 | 17.05 | LOC105371017 | uncharacterized LOC105371017 | | ncRNA |
| 1.03E+08 | 0.999946 | 0.0148 | 1.041 | -2.43727 | -2.53686 | 17.92 | LOC102723753 | HECT and RLD domain containing E3 ubiquitin protein ligase 2 pseudogene | | pseudo |
| 26851 | 0.999946 | 0.0113 | 0.642 | -2.5332 | -1.62631 | 72.52 | SNORD3B-1 | small nucleolar RNA, C/D box 3B-1 | RNU3A1\|U3a\|U3b1\|U3b2 | snoRNA |
| 1.05E+08 | 0.999946 | 0.0207 | 0.814 | -2.31358 | -1.88331 | 21.1 | LOC105372621 | uncharacterized LOC105372621 | | ncRNA |
| 55816 | 0.999946 | 0.0271 | 0.879 | -2.2105 | -1.94219 | 13.67 | DOK5 | docking protein 5 | C20orf180\|IRS-6\|IRS6 | protein-coding |
| 1.05E+08 | 0.999946 | 0.0138 | 0.765 | -2.46238 | -1.88403 | 11.62 | LOC105372777 | uncharacterized LOC105372777 | | ncRNA |
| 1.06E+08 | 0.999946 | 0.0107 | 0.681 | -2.55073 | -1.73769 | 12.11 | BMS1P22 | BMS1 pseudogene 22 | | pseudo |
| 728441 | 0.999946 | 0.0321 | 0.887 | -2.14368 | -1.90075 | 61.6 | GGT2P | gamma-glutamyltransferase 2, pseudogene | GGT\|GGT 2\|GGT2 | pseudo |
| 1.01E+08 | 0.999946 | 0.0266 | 0.724 | -2.2168 | -1.60567 | 14.16 | MIR4762 | microRNA 4762 | | ncRNA |
| 1.03E+08 | 0.999946 | 0.0122 | 0.933 | -2.50532 | -2.33845 | 67.05 | LOC102724197 | inactive glutathione hydrolase 2 | GGT2 | protein-coding |
| 1.03E+08 | 0.999946 | 0.0208 | 0.952 | -2.31221 | -2.20071 | 44.45 | LOC102724823 | replaced by ID 2679 | |  |
| 139411 | 0.999946 | 0.0201 | 0.656 | -2.32398 | -1.52523 | 69.31 | CFTR | patched domain containing 1 | AUTSX4 | protein-coding |
| 1.05E+08 | NA | 0.012 | 0.864 | -2.51215 | -2.16961 | 9.11 | LINC01946 | long intergenic non-protein coding RNA 1946 | | ncRNA |
| 1.05E+08 | NA | 0.0415 | 0.862 | -2.03842 | -1.75663 | 7.92 | LOC105369685 | uncharacterized LOC105369685 | | ncRNA |
| 1.05E+08 | NA | 0.00905 | 0.919 | -2.61007 | -2.3981 | 8.37 | LOC105372209 | uncharacterized LOC105372209 | | ncRNA |
