# Supplementary material for: Differential expression of ion channel coding genes in the endometrium of women experiencing recurrent implantation failures
Source: Sci Rep. 2024 Aug 27;14:19822. doi: 10.1038/s41598-024-70778-9 (PMC11349755; doi:10.1038/s41598-024-70778-9)
Supplement: Supplementary file 2 — Supplementary Information 2. [file 41598_2024_70778_MOESM2_ESM.docx]

| #clustering method | cluster number | cluster color | gene count | protein name | protein identifier | protein description |
| --- | --- | --- | --- | --- | --- | --- |
| kmeans | 1 | Red | 8 | NDFIP1 | 9606.ENSP00000253814 | NEDD4 family-interacting protein 1; Activates HECT domain-containing E3 ubiquitin-protein ligases, including NEDD4 and ITCH, and consequently modulates the stability of their targets. As a result, controls many cellular processes. Prevents chronic T-helper cell-mediated inflammation by activating ITCH and thus controlling JUNB degradation (By similarity). Promotes pancreatic beta cell death through degradation of JUNB and inhibition of the unfolded protein response, leading to reduction of insulin secretion. Restricts the production of proinflammatory cytokines in effector Th17 T-cells [...] |
| kmeans | 1 | Red | 8 | NEDD4 | 9606.ENSP00000424827 | E3 ubiquitin-protein ligase NEDD4; E3 ubiquitin-protein ligase which accepts ubiquitin from an E2 ubiquitin-conjugating enzyme in the form of a thioester and then directly transfers the ubiquitin to targeted substrates. Specifically ubiquitinates 'Lys-63' in target proteins. Involved in the pathway leading to the degradation of VEGFR-2/KDFR, independently of its ubiquitin-ligase activity. Monoubiquitinates IGF1R at multiple sites, thus leading to receptor internalization and degradation in lysosomes. Ubiquitinates FGFR1, leading to receptor internalization and degradation in lysosomes. [...] |
| kmeans | 1 | Red | 8 | NEDD4L | 9606.ENSP00000383199 | E3 ubiquitin-protein ligase NEDD4-like; E3 ubiquitin-protein ligase which accepts ubiquitin from an E2 ubiquitin-conjugating enzyme in the form of a thioester and then directly transfers the ubiquitin to targeted substrates. Inhibits TGF- beta signaling by triggering SMAD2 and TGFBR1 ubiquitination and proteasome-dependent degradation. Promotes ubiquitination and internalization of various plasma membrane channels such as ENaC, SCN2A/Nav1.2, SCN3A/Nav1.3, SCN5A/Nav1.5, SCN9A/Nav1.7, SCN10A/Nav1.8, KCNA3/Kv1.3, KCNH2, EAAT1, KCNQ2/Kv7.2, KCNQ3/Kv7.3 or CLC5. Promotes ubiquitination and [...] |
| kmeans | 1 | Red | 8 | SCNN1A | 9606.ENSP00000353292 | Amiloride-sensitive sodium channel subunit alpha; Sodium permeable non-voltage-sensitive ion channel inhibited by the diuretic amiloride. Mediates the electrodiffusion of the luminal sodium (and water, which follows osmotically) through the apical membrane of epithelial cells. Plays an essential role in electrolyte and blood pressure homeostasis, but also in airway surface liquid homeostasis, which is important for proper clearance of mucus. Controls the reabsorption of sodium in kidney, colon, lung and eccrine sweat glands. Also plays a role in taste perception. |
| kmeans | 1 | Red | 8 | SCNN1B | 9606.ENSP00000345751 | Amiloride-sensitive sodium channel subunit beta; Sodium permeable non-voltage-sensitive ion channel inhibited by the diuretic amiloride. Mediates the electrodiffusion of the luminal sodium (and water, which follows osmotically) through the apical membrane of epithelial cells. Plays an essential role in electrolyte and blood pressure homeostasis, but also in airway surface liquid homeostasis, which is important for proper clearance of mucus. Controls the reabsorption of sodium in kidney, colon, lung and sweat glands. Also plays a role in taste perception; Belongs to the amiloride-sensit [...] |
| kmeans | 1 | Red | 8 | SCNN1G | 9606.ENSP00000300061 | Amiloride-sensitive sodium channel subunit gamma; Sodium permeable non-voltage-sensitive ion channel inhibited by the diuretic amiloride. Mediates the electrodiffusion of the luminal sodium (and water, which follows osmotically) through the apical membrane of epithelial cells. Plays an essential role in electrolyte and blood pressure homeostasis, but also in airway surface liquid homeostasis, which is important for proper clearance of mucus. Controls the reabsorption of sodium in kidney, colon, lung and sweat glands. Also plays a role in taste perception. |
| kmeans | 1 | Red | 8 | SMAD3 | 9606.ENSP00000332973 | Mothers against decapentaplegic homolog 3; Receptor-regulated SMAD (R-SMAD) that is an intracellular signal transducer and transcriptional modulator activated by TGF-beta (transforming growth factor) and activin type 1 receptor kinases. Binds the TRE element in the promoter region of many genes that are regulated by TGF-beta and, on formation of the SMAD3/SMAD4 complex, activates transcription. Also can form a SMAD3/SMAD4/JUN/FOS complex at the AP- 1/SMAD site to regulate TGF-beta-mediated transcription. Has an inhibitory effect on wound healing probably by modulating both growth and m [...] |
| kmeans | 1 | Red | 8 | SMAD7 | 9606.ENSP00000262158 | Mothers against decapentaplegic homolog 7; Antagonist of signaling by TGF-beta (transforming growth factor) type 1 receptor superfamily members; has been shown to inhibit TGF-beta (Transforming growth factor) and activin signaling by associating with their receptors thus preventing SMAD2 access. Functions as an adapter to recruit SMURF2 to the TGF-beta receptor complex. Also acts by recruiting the PPP1R15A-PP1 complex to TGFBR1, which promotes its dephosphorylation. Positively regulates PDPK1 kinase activity by stimulating its dissociation from the 14-3-3 protein YWHAQ which acts as a [...] |
| kmeans | 2 | Yellow | 8 | CACNA1C | 9606.ENSP00000266376 | Voltage-dependent L-type calcium channel subunit alpha-1C; Pore-forming, alpha-1C subunit of the voltage-gated calcium channel that gives rise to L-type calcium currents. Mediates influx of calcium ions into the cytoplasm, and thereby triggers calcium release from the sarcoplasm (By similarity). Plays an important role in excitation-contraction coupling in the heart. Required for normal heart development and normal regulation of heart rhythm. Required for normal contraction of smooth muscle cells in blood vessels and in the intestine. Essential for normal blood pressure regulation via [...] |
| kmeans | 2 | Yellow | 8 | CACNA1H | 9606.ENSP00000334198 | Voltage-dependent T-type calcium channel subunit alpha-1H; Voltage-sensitive calcium channel that gives rise to T-type calcium currents. T-type calcium channels belong to the 'low-voltage activated (LVA)' group. A particularity of this type of channel is an opening at quite negative potentials, and a voltage-dependent inactivation. T-type channels serve pacemaking functions in both central neurons and cardiac nodal cells and support calcium signaling in secretory cells and vascular smooth muscle (Probable). They may also be involved in the modulation of firing patterns of neurons. In t [...] |
| kmeans | 2 | Yellow | 8 | CALM3 | 9606.ENSP00000291295 | Calmodulin-1; Calmodulin mediates the control of a large number of enzymes, ion channels, aquaporins and other proteins through calcium-binding. Among the enzymes to be stimulated by the calmodulin-calcium complex are a number of protein kinases and phosphatases. Together with CCP110 and centrin, is involved in a genetic pathway that regulates the centrosome cycle and progression through cytokinesis. Mediates calcium-dependent inactivation of CACNA1C. Positively regulates calcium-activated potassium channel activity of KCNN2. |
| kmeans | 2 | Yellow | 8 | CAMKK2 | 9606.ENSP00000312741 | Calcium/calmodulin-dependent protein kinase kinase 2; Calcium/calmodulin-dependent protein kinase belonging to a proposed calcium-triggered signaling cascade involved in a number of cellular processes. Isoform 1, isoform 2 and isoform 3 phosphorylate CAMK1 and CAMK4. Isoform 3 phosphorylates CAMK1D. Isoform 4, isoform 5 and isoform 6 lacking part of the calmodulin-binding domain are inactive. Efficiently phosphorylates 5'-AMP-activated protein kinase (AMPK) trimer, including that consisting of PRKAA1, PRKAB1 and PRKAG1. This phosphorylation is stimulated in response to Ca(2+) signals ( [...] |
| kmeans | 2 | Yellow | 8 | NOS2 | 9606.ENSP00000327251 | Nitric oxide synthase, inducible; Produces nitric oxide (NO) which is a messenger molecule with diverse functions throughout the body. In macrophages, NO mediates tumoricidal and bactericidal actions. Also has nitrosylase activity and mediates cysteine S-nitrosylation of cytoplasmic target proteins such PTGS2/COX2 (By similarity). As component of the iNOS-S100A8/9 transnitrosylase complex involved in the selective inflammatory stimulus-dependent S-nitrosylation of GAPDH on 'Cys-247' implicated in regulation of the GAIT complex activity and probably multiple targets including ANXA5, EZR [...] |
| kmeans | 2 | Yellow | 8 | NOS3 | 9606.ENSP00000297494 | Nitric oxide synthase, endothelial; Produces nitric oxide (NO) which is implicated in vascular smooth muscle relaxation through a cGMP-mediated signal transduction pathway. NO mediates vascular endothelial growth factor (VEGF)-induced angiogenesis in coronary vessels and promotes blood clotting through the activation of platelets; Belongs to the NOS family. |
| kmeans | 2 | Yellow | 8 | RYR1 | 9606.ENSP00000352608 | Ryanodine receptor 1; Calcium channel that mediates the release of Ca(2+) from the sarcoplasmic reticulum into the cytoplasm and thereby plays a key role in triggering muscle contraction following depolarization of T-tubules. Repeated very high-level exercise increases the open probability of the channel and leads to Ca(2+) leaking into the cytoplasm. Can also mediate the release of Ca(2+) from intracellular stores in neurons, and may thereby promote prolonged Ca(2+) signaling in the brain. Required for normal embryonic development of muscle fibers and skeletal muscle. Required for nor [...] |
| kmeans | 2 | Yellow | 8 | SCN5A | 9606.ENSP00000328968 | Sodium channel protein type 5 subunit alpha; This protein mediates the voltage-dependent sodium ion permeability of excitable membranes. Assuming opened or closed conformations in response to the voltage difference across the membrane, the protein forms a sodium-selective channel through which Na(+) ions may pass in accordance with their electrochemical gradient. It is a tetrodotoxin-resistant Na(+) channel isoform. This channel is responsible for the initial upstroke of the action potential. Channel inactivation is regulated by intracellular calcium levels. Belongs to the sodium chann [...] |
| kmeans | 3 | Green | 6 | AKAP9 | 9606.ENSP00000348573 | A-kinase anchor protein 9; Scaffolding protein that assembles several protein kinases and phosphatases on the centrosome and Golgi apparatus. Required to maintain the integrity of the Golgi apparatus. Required for microtubule nucleation at the cis-side of the Golgi apparatus. Required for association of the centrosomes with the poles of the bipolar mitotic spindle during metaphase. In complex with PDE4DIP isoform 13/MMG8/SMYLE, recruits CAMSAP2 to the Golgi apparatus and tethers non-centrosomal minus-end microtubules to the Golgi, an important step for polarized cell movement. In compl [...] |
| kmeans | 3 | Green | 6 | KCNE1 | 9606.ENSP00000382226 | Potassium voltage-gated channel subfamily E member 1; Ancillary protein that assembles as a beta subunit with a voltage-gated potassium channel complex of pore-forming alpha subunits. Modulates the gating kinetics and enhances stability of the channel complex. Assembled with KCNB1 modulates the gating characteristics of the delayed rectifier voltage-dependent potassium channel KCNB1. Assembled with KCNQ1/KVLQT1 is proposed to form the slowly activating delayed rectifier cardiac potassium (IKs) channel. The outward current reaches its steady state only after 50 seconds. Assembled with K [...] |
| kmeans | 3 | Green | 6 | KCNE2 | 9606.ENSP00000290310 | Potassium voltage-gated channel subfamily E member 2; Ancillary protein that assembles as a beta subunit with a voltage-gated potassium channel complex of pore-forming alpha subunits. Modulates the gating kinetics and enhances stability of the channel complex. Assembled with KCNB1 modulates the gating characteristics of the delayed rectifier voltage-dependent potassium channel KCNB1. Associated with KCNH2/HERG is proposed to form the rapidly activating component of the delayed rectifying potassium current in heart (IKr). May associate with KCNQ2 and/or KCNQ3 and modulate the native M-t [...] |
| kmeans | 3 | Green | 6 | KCNE3 | 9606.ENSP00000310557 | Potassium voltage-gated channel subfamily E member 3; Ancillary protein that assembles as a beta subunit with a voltage-gated potassium channel complex of pore-forming alpha subunits. Modulates the gating kinetics and enhances stability of the channel complex. Assembled with KCNB1 modulates the gating characteristics of the delayed rectifier voltage-dependent potassium channel KCNB1. Associated with KCNC4/Kv3.4 is proposed to form the subthreshold voltage-gated potassium channel in skeletal muscle and to establish the resting membrane potential (RMP) in muscle cells. Associated with KC [...] |
| kmeans | 3 | Green | 6 | KCNJ2 | 9606.ENSP00000243457 | Inward rectifier potassium channel 2; Probably participates in establishing action potential waveform and excitability of neuronal and muscle tissues. Inward rectifier potassium channels are characterized by a greater tendency to allow potassium to flow into the cell rather than out of it. Their voltage dependence is regulated by the concentration of extracellular potassium; as external potassium is raised, the voltage range of the channel opening shifts to more positive voltages. The inward rectification is mainly due to the blockage of outward current by internal magnesium. Can be bl [...] |
| kmeans | 3 | Green | 6 | KCNQ1 | 9606.ENSP00000155840 | Potassium voltage-gated channel subfamily KQT member 1; Potassium channel that plays an important role in a number of tissues, including heart, inner ear, stomach and colon (By similarity). Associates with KCNE beta subunits that modulates current kinetics (By similarity). Induces a voltage-dependent by rapidly activating and slowly deactivating potassium-selective outward current (By similarity). Promotes also a delayed voltage activated potassium current showing outward rectification characteristic (By similarity). During beta-adrenergic receptor stimulation participates in cardiac r [...] |
| kmeans | 4 | Blue | 4 | CFTR | 9606.ENSP00000003084 | Cystic fibrosis transmembrane conductance regulator; Epithelial ion channel that plays an important role in the regulation of epithelial ion and water transport and fluid homeostasis. Mediates the transport of chloride ions across the cell membrane. Channel activity is coupled to ATP hydrolysis. The ion channel is also permeable to HCO(3-); selectivity depends on the extracellular chloride concentration. Exerts its function also by modulating the activity of other ion channels and transporters. Plays an important role in airway fluid homeostasis. Contributes to the regulation of the pH [...] |
| kmeans | 4 | Blue | 4 | EZR | 9606.ENSP00000338934 | Ezrin; Probably involved in connections of major cytoskeletal structures to the plasma membrane. In epithelial cells, required for the formation of microvilli and membrane ruffles on the apical pole. Along with PLEKHG6, required for normal macropinocytosis. |
| kmeans | 4 | Blue | 4 | GOPC | 9606.ENSP00000357484 | Golgi-associated PDZ and coiled-coil motif-containing protein; Plays a role in intracellular protein trafficking and degradation. May regulate CFTR chloride currents and acid-induced ASIC3 currents by modulating cell surface expression of both channels. May also regulate the intracellular trafficking of the ADR1B receptor. May play a role in autophagy. Overexpression results in CFTR intracellular retention and degradation in the lysosomes. |
| kmeans | 4 | Blue | 4 | SLC9A3R1 | 9606.ENSP00000262613 | Na(+)/H(+) exchange regulatory cofactor NHE-RF1; Scaffold protein that connects plasma membrane proteins with members of the ezrin/moesin/radixin family and thereby helps to link them to the actin cytoskeleton and to regulate their surface expression. Necessary for recycling of internalized ADRB2. Was first known to play a role in the regulation of the activity and subcellular location of SLC9A3. Necessary for cAMP-mediated phosphorylation and inhibition of SLC9A3. May enhance Wnt signaling. May participate in HTR4 targeting to microvilli (By similarity). Involved in the regulation of [...] |
