# Supplementary material for: Differential expression of ion channel coding genes in the endometrium of women experiencing recurrent implantation failures
Source: Sci Rep. 2024 Aug 27;14:19822. doi: 10.1038/s41598-024-70778-9 (PMC11349755; doi:10.1038/s41598-024-70778-9)
Supplement: Supplementary file 3 — Supplementary Information 3. [file 41598_2024_70778_MOESM3_ESM.docx]

Sheet 1

| Enrichment FDR | nGenes | Pathway Genes | Fold Enrichment | Pathway | URL | Genes |
| --- | --- | --- | --- | --- | --- | --- |
| 2.00E-35 | 14 | 26 | 511.4487 | Sodium channel complex | http://amigo.geneontology.org/amigo/term/GO:0034706 | SCN4A CACNA1I SCN1B SCNN1A TRPM4 SCN7A GRIK4 SCN2B SCNN1D SCN3B SCNN1G SCNN1B SCN4B CACNA1H |
| 2.03E-19 | 8 | 17 | 446.9804 | Voltage-gated sodium channel complex | http://amigo.geneontology.org/amigo/term/GO:0001518 | SCN4A CACNA1I SCN1B SCN7A SCN2B SCN3B SCN4B CACNA1H |
| 3.38E-25 | 16 | 238 | 63.85434 | Cation channel complex | http://amigo.geneontology.org/amigo/term/GO:0034703 | SCN4A CACNA1I SCN1B SCNN1A TRPM4 SCN7A GRIK4 SCN2B KCNJ1 SCNN1D SCN3B SCNN1G SCNN1B SCN4B CACNA1H KCNE1B |
| 3.38E-25 | 17 | 324 | 49.83693 | Ion channel complex | http://amigo.geneontology.org/amigo/term/GO:0034702 | CFTR SCN4A CACNA1I SCN1B SCNN1A TRPM4 SCN7A GRIK4 SCN2B KCNJ1 SCNN1D SCN3B SCNN1G SCNN1B SCN4B CACNA1H KCNE1B |
| 0.009222 | 2 | 47 | 40.41844 | Voltage-gated calcium channel complex | http://amigo.geneontology.org/amigo/term/GO:0005891 | CACNA1I CACNA1H |
| 2.98E-23 | 17 | 427 | 37.81538 | Transmembrane transporter complex | http://amigo.geneontology.org/amigo/term/GO:1902495 | CFTR SCN4A CACNA1I SCN1B SCNN1A TRPM4 SCN7A GRIK4 SCN2B KCNJ1 SCNN1D SCN3B SCNN1G SCNN1B SCN4B CACNA1H KCNE1B |
| 4.32E-23 | 17 | 442 | 36.53205 | Transporter complex | http://amigo.geneontology.org/amigo/term/GO:1990351 | CFTR SCN4A CACNA1I SCN1B SCNN1A TRPM4 SCN7A GRIK4 SCN2B KCNJ1 SCNN1D SCN3B SCNN1G SCNN1B SCN4B CACNA1H KCNE1B |
| 0.011394 | 2 | 54 | 35.17901 | Intercalated disc | http://amigo.geneontology.org/amigo/term/GO:0014704 | SCN1B SCN4B |
| 0.001542 | 3 | 104 | 27.39904 | Potassium channel complex | http://amigo.geneontology.org/amigo/term/GO:0034705 | GRIK4 KCNJ1 KCNE1B |
| 0.019835 | 2 | 78 | 24.3547 | Calcium channel complex | http://amigo.geneontology.org/amigo/term/GO:0034704 | CACNA1I CACNA1H |
| 0.019835 | 2 | 80 | 23.74583 | Cell-cell contact zone | http://amigo.geneontology.org/amigo/term/GO:0044291 | SCN1B SCN4B |
| 5.45E-13 | 13 | 712 | 17.34246 | Plasma membrane protein complex | http://amigo.geneontology.org/amigo/term/GO:0098797 | SCN4A CACNA1I SCN1B SCN7A GRIK4 SCN2B KCNJ1 SCN3B SCNN1G SCNN1B SCN4B CACNA1H KCNE1B |
| 2.62E-05 | 6 | 385 | 14.8026 | Apical plasma membrane | http://amigo.geneontology.org/amigo/term/GO:0016324 | CFTR SLC12A3 SCNN1A SCNN1G SCNN1B KCNE1B |
| 6.11E-05 | 6 | 453 | 12.58057 | Apical part of cell | http://amigo.geneontology.org/amigo/term/GO:0045177 | CFTR SLC12A3 SCNN1A SCNN1G SCNN1B KCNE1B |
| 1.11E-14 | 17 | 1415 | 11.41143 | Membrane protein complex | http://amigo.geneontology.org/amigo/term/GO:0098796 | CFTR SCN4A CACNA1I SCN1B SCNN1A TRPM4 SCN7A GRIK4 SCN2B KCNJ1 SCNN1D SCN3B SCNN1G SCNN1B SCN4B CACNA1H KCNE1B |
| 3.75E-14 | 18 | 1881 | 9.089314 | Integral component of plasma membrane | http://amigo.geneontology.org/amigo/term/GO:0005887 | CFTR SCN4A SLC12A3 CACNA1I SCN1B SCNN1A TRPM4 SCN7A GRIK4 SCN2B KCNJ1 SCNN1D SCN3B SCNN1G SCNN1B SCN4B CACNA1H KCNE1B |
| 0.014333 | 4 | 519 | 7.320488 | Cell-cell junction | http://amigo.geneontology.org/amigo/term/GO:0005911 | SCN1B WNK4 SCN4B WNK3 |
| 0.000383 | 8 | 1323 | 5.743512 | Plasma membrane region | http://amigo.geneontology.org/amigo/term/GO:0098590 | CFTR NEDD4 SLC12A3 SCNN1A GRIK4 SCNN1G SCNN1B KCNE1B |
| 0.019835 | 6 | 1444 | 3.946676 | Neuron projection | http://amigo.geneontology.org/amigo/term/GO:0043005 | SCN4A NEDD4 CACNA1I SCN1B SCN7A CACNA1H |

Sheet 2

| Enrichment FDR | nGenes | Pathway Genes | Fold Enrichment | Pathway | URL | Genes |
| --- | --- | --- | --- | --- | --- | --- |
| 2.91E-18 | 8 | 24 | 316.6111 | Voltage-gated sodium channel activity | http://amigo.geneontology.org/amigo/term/GO:0005248 | SCN4A CACNA1I SCN1B SCN7A SCN2B SCN3B SCN4B CACNA1H |
| 5.12E-28 | 13 | 46 | 268.4312 | Sodium channel activity | http://amigo.geneontology.org/amigo/term/GO:0005272 | SCN4A CACNA1I SCN1B SCNN1A SCN7A GRIK4 SCN2B SCNN1D SCN3B SCNN1G SCNN1B SCN4B CACNA1H |
| 6.29E-17 | 8 | 34 | 223.4902 | Ion channel inhibitor activity | http://amigo.geneontology.org/amigo/term/GO:0008200 | NEDD4L WNK1 NEDD4 SCN1B WNK4 WNK2 SCN3B WNK3 |
| 7.29E-17 | 8 | 35 | 217.1048 | Channel inhibitor activity | http://amigo.geneontology.org/amigo/term/GO:0016248 | NEDD4L WNK1 NEDD4 SCN1B WNK4 WNK2 SCN3B WNK3 |
| 1.86E-23 | 14 | 153 | 86.91285 | Sodium ion transmembrane transporter activity | http://amigo.geneontology.org/amigo/term/GO:0015081 | SCN4A SLC12A3 CACNA1I SCN1B SCNN1A SCN7A GRIK4 SCN2B SCNN1D SCN3B SCNN1G SCNN1B SCN4B CACNA1H |
| 7.90E-20 | 12 | 145 | 78.6069 | Ion channel regulator activity | http://amigo.geneontology.org/amigo/term/GO:0099106 | CFTR NEDD4L WNK1 NEDD4 SCN1B WNK4 SCN2B WNK2 SCN3B SCN4B WNK3 KCNE1B |
| 1.21E-19 | 12 | 151 | 75.48344 | Channel regulator activity | http://amigo.geneontology.org/amigo/term/GO:0016247 | CFTR NEDD4L WNK1 NEDD4 SCN1B WNK4 SCN2B WNK2 SCN3B SCN4B WNK3 KCNE1B |
| 4.79E-14 | 10 | 211 | 45.0158 | Voltage-gated ion channel activity | http://amigo.geneontology.org/amigo/term/GO:0005244 | SCN4A CACNA1I SCN1B SCN7A SCN2B KCNJ1 SCN3B SCN4B CACNA1H KCNE1B |
| 4.73E-24 | 17 | 365 | 44.23881 | Gated channel activity | http://amigo.geneontology.org/amigo/term/GO:0022836 | CFTR SCN4A CACNA1I SCN1B SCNN1A TRPM4 SCN7A GRIK4 SCN2B KCNJ1 SCNN1D SCN3B SCNN1G SCNN1B SCN4B CACNA1H KCNE1B |
| 1.09E-22 | 16 | 350 | 43.42095 | Cation channel activity | http://amigo.geneontology.org/amigo/term/GO:0005261 | SCN4A CACNA1I SCN1B SCNN1A TRPM4 SCN7A GRIK4 SCN2B KCNJ1 SCNN1D SCN3B SCNN1G SCNN1B SCN4B CACNA1H KCNE1B |
| 9.79E-23 | 17 | 454 | 35.56645 | Metal ion transmembrane transporter activity | http://amigo.geneontology.org/amigo/term/GO:0046873 | SCN4A SLC12A3 CACNA1I SCN1B SCNN1A TRPM4 SCN7A GRIK4 SCN2B KCNJ1 SCNN1D SCN3B SCNN1G SCNN1B SCN4B CACNA1H KCNE1B |
| 9.79E-23 | 17 | 459 | 35.17901 | Ion channel activity | http://amigo.geneontology.org/amigo/term/GO:0005216 | CFTR SCN4A CACNA1I SCN1B SCNN1A TRPM4 SCN7A GRIK4 SCN2B KCNJ1 SCNN1D SCN3B SCNN1G SCNN1B SCN4B CACNA1H KCNE1B |
| 3.60E-22 | 17 | 509 | 31.72331 | Channel activity | http://amigo.geneontology.org/amigo/term/GO:0015267 | CFTR SCN4A CACNA1I SCN1B SCNN1A TRPM4 SCN7A GRIK4 SCN2B KCNJ1 SCNN1D SCN3B SCNN1G SCNN1B SCN4B CACNA1H KCNE1B |
| 3.60E-22 | 17 | 509 | 31.72331 | Passive transmembrane transporter activity | http://amigo.geneontology.org/amigo/term/GO:0022803 | CFTR SCN4A CACNA1I SCN1B SCNN1A TRPM4 SCN7A GRIK4 SCN2B KCNJ1 SCNN1D SCN3B SCNN1G SCNN1B SCN4B CACNA1H KCNE1B |
| 1.29E-20 | 17 | 636 | 25.38863 | Inorganic cation transmembrane transporter activity | http://amigo.geneontology.org/amigo/term/GO:0022890 | SCN4A SLC12A3 CACNA1I SCN1B SCNN1A TRPM4 SCN7A GRIK4 SCN2B KCNJ1 SCNN1D SCN3B SCNN1G SCNN1B SCN4B CACNA1H KCNE1B |
| 5.06E-20 | 17 | 693 | 23.30038 | Cation transmembrane transporter activity | http://amigo.geneontology.org/amigo/term/GO:0008324 | SCN4A SLC12A3 CACNA1I SCN1B SCNN1A TRPM4 SCN7A GRIK4 SCN2B KCNJ1 SCNN1D SCN3B SCNN1G SCNN1B SCN4B CACNA1H KCNE1B |
| 5.02E-21 | 18 | 771 | 22.1751 | Inorganic molecular entity transmembrane transporter activity | http://amigo.geneontology.org/amigo/term/GO:0015318 | CFTR SCN4A SLC12A3 CACNA1I SCN1B SCNN1A TRPM4 SCN7A GRIK4 SCN2B KCNJ1 SCNN1D SCN3B SCNN1G SCNN1B SCN4B CACNA1H KCNE1B |
| 7.26E-20 | 18 | 909 | 18.80858 | Ion transmembrane transporter activity | http://amigo.geneontology.org/amigo/term/GO:0015075 | CFTR SCN4A SLC12A3 CACNA1I SCN1B SCNN1A TRPM4 SCN7A GRIK4 SCN2B KCNJ1 SCNN1D SCN3B SCNN1G SCNN1B SCN4B CACNA1H KCNE1B |
| 1.57E-17 | 18 | 1248 | 13.69952 | Transmembrane transporter activity | http://amigo.geneontology.org/amigo/term/GO:0022857 | CFTR SCN4A SLC12A3 CACNA1I SCN1B SCNN1A TRPM4 SCN7A GRIK4 SCN2B KCNJ1 SCNN1D SCN3B SCNN1G SCNN1B SCN4B CACNA1H KCNE1B |
| 6.76E-17 | 18 | 1364 | 12.53446 | Transporter activity | http://amigo.geneontology.org/amigo/term/GO:0005215 | CFTR SCN4A SLC12A3 CACNA1I SCN1B SCNN1A TRPM4 SCN7A GRIK4 SCN2B KCNJ1 SCNN1D SCN3B SCNN1G SCNN1B SCN4B CACNA1H KCNE1B |

Sheet 3

| Enrichment FDR | nGenes | Pathway Genes | Fold Enrichment | Pathway | URL | Genes |
| --- | --- | --- | --- | --- | --- | --- |
| 2.91E-18 | 8 | 24 | 316.6111 | Voltage-gated sodium channel activity | http://amigo.geneontology.org/amigo/term/GO:0005248 | SCN4A CACNA1I SCN1B SCN7A SCN2B SCN3B SCN4B CACNA1H |
| 5.12E-28 | 13 | 46 | 268.4312 | Sodium channel activity | http://amigo.geneontology.org/amigo/term/GO:0005272 | SCN4A CACNA1I SCN1B SCNN1A SCN7A GRIK4 SCN2B SCNN1D SCN3B SCNN1G SCNN1B SCN4B CACNA1H |
| 6.29E-17 | 8 | 34 | 223.4902 | Ion channel inhibitor activity | http://amigo.geneontology.org/amigo/term/GO:0008200 | NEDD4L WNK1 NEDD4 SCN1B WNK4 WNK2 SCN3B WNK3 |
| 7.29E-17 | 8 | 35 | 217.1048 | Channel inhibitor activity | http://amigo.geneontology.org/amigo/term/GO:0016248 | NEDD4L WNK1 NEDD4 SCN1B WNK4 WNK2 SCN3B WNK3 |
| 1.86E-23 | 14 | 153 | 86.91285 | Sodium ion transmembrane transporter activity | http://amigo.geneontology.org/amigo/term/GO:0015081 | SCN4A SLC12A3 CACNA1I SCN1B SCNN1A SCN7A GRIK4 SCN2B SCNN1D SCN3B SCNN1G SCNN1B SCN4B CACNA1H |
| 7.90E-20 | 12 | 145 | 78.6069 | Ion channel regulator activity | http://amigo.geneontology.org/amigo/term/GO:0099106 | CFTR NEDD4L WNK1 NEDD4 SCN1B WNK4 SCN2B WNK2 SCN3B SCN4B WNK3 KCNE1B |
| 1.21E-19 | 12 | 151 | 75.48344 | Channel regulator activity | http://amigo.geneontology.org/amigo/term/GO:0016247 | CFTR NEDD4L WNK1 NEDD4 SCN1B WNK4 SCN2B WNK2 SCN3B SCN4B WNK3 KCNE1B |
| 4.79E-14 | 10 | 211 | 45.0158 | Voltage-gated ion channel activity | http://amigo.geneontology.org/amigo/term/GO:0005244 | SCN4A CACNA1I SCN1B SCN7A SCN2B KCNJ1 SCN3B SCN4B CACNA1H KCNE1B |
| 4.73E-24 | 17 | 365 | 44.23881 | Gated channel activity | http://amigo.geneontology.org/amigo/term/GO:0022836 | CFTR SCN4A CACNA1I SCN1B SCNN1A TRPM4 SCN7A GRIK4 SCN2B KCNJ1 SCNN1D SCN3B SCNN1G SCNN1B SCN4B CACNA1H KCNE1B |
| 1.09E-22 | 16 | 350 | 43.42095 | Cation channel activity | http://amigo.geneontology.org/amigo/term/GO:0005261 | SCN4A CACNA1I SCN1B SCNN1A TRPM4 SCN7A GRIK4 SCN2B KCNJ1 SCNN1D SCN3B SCNN1G SCNN1B SCN4B CACNA1H KCNE1B |
| 9.79E-23 | 17 | 454 | 35.56645 | Metal ion transmembrane transporter activity | http://amigo.geneontology.org/amigo/term/GO:0046873 | SCN4A SLC12A3 CACNA1I SCN1B SCNN1A TRPM4 SCN7A GRIK4 SCN2B KCNJ1 SCNN1D SCN3B SCNN1G SCNN1B SCN4B CACNA1H KCNE1B |
| 9.79E-23 | 17 | 459 | 35.17901 | Ion channel activity | http://amigo.geneontology.org/amigo/term/GO:0005216 | CFTR SCN4A CACNA1I SCN1B SCNN1A TRPM4 SCN7A GRIK4 SCN2B KCNJ1 SCNN1D SCN3B SCNN1G SCNN1B SCN4B CACNA1H KCNE1B |
| 3.60E-22 | 17 | 509 | 31.72331 | Channel activity | http://amigo.geneontology.org/amigo/term/GO:0015267 | CFTR SCN4A CACNA1I SCN1B SCNN1A TRPM4 SCN7A GRIK4 SCN2B KCNJ1 SCNN1D SCN3B SCNN1G SCNN1B SCN4B CACNA1H KCNE1B |
| 3.60E-22 | 17 | 509 | 31.72331 | Passive transmembrane transporter activity | http://amigo.geneontology.org/amigo/term/GO:0022803 | CFTR SCN4A CACNA1I SCN1B SCNN1A TRPM4 SCN7A GRIK4 SCN2B KCNJ1 SCNN1D SCN3B SCNN1G SCNN1B SCN4B CACNA1H KCNE1B |
| 1.29E-20 | 17 | 636 | 25.38863 | Inorganic cation transmembrane transporter activity | http://amigo.geneontology.org/amigo/term/GO:0022890 | SCN4A SLC12A3 CACNA1I SCN1B SCNN1A TRPM4 SCN7A GRIK4 SCN2B KCNJ1 SCNN1D SCN3B SCNN1G SCNN1B SCN4B CACNA1H KCNE1B |
| 5.06E-20 | 17 | 693 | 23.30038 | Cation transmembrane transporter activity | http://amigo.geneontology.org/amigo/term/GO:0008324 | SCN4A SLC12A3 CACNA1I SCN1B SCNN1A TRPM4 SCN7A GRIK4 SCN2B KCNJ1 SCNN1D SCN3B SCNN1G SCNN1B SCN4B CACNA1H KCNE1B |
| 5.02E-21 | 18 | 771 | 22.1751 | Inorganic molecular entity transmembrane transporter activity | http://amigo.geneontology.org/amigo/term/GO:0015318 | CFTR SCN4A SLC12A3 CACNA1I SCN1B SCNN1A TRPM4 SCN7A GRIK4 SCN2B KCNJ1 SCNN1D SCN3B SCNN1G SCNN1B SCN4B CACNA1H KCNE1B |
| 7.26E-20 | 18 | 909 | 18.80858 | Ion transmembrane transporter activity | http://amigo.geneontology.org/amigo/term/GO:0015075 | CFTR SCN4A SLC12A3 CACNA1I SCN1B SCNN1A TRPM4 SCN7A GRIK4 SCN2B KCNJ1 SCNN1D SCN3B SCNN1G SCNN1B SCN4B CACNA1H KCNE1B |
| 1.57E-17 | 18 | 1248 | 13.69952 | Transmembrane transporter activity | http://amigo.geneontology.org/amigo/term/GO:0022857 | CFTR SCN4A SLC12A3 CACNA1I SCN1B SCNN1A TRPM4 SCN7A GRIK4 SCN2B KCNJ1 SCNN1D SCN3B SCNN1G SCNN1B SCN4B CACNA1H KCNE1B |
| 6.76E-17 | 18 | 1364 | 12.53446 | Transporter activity | http://amigo.geneontology.org/amigo/term/GO:0005215 | CFTR SCN4A SLC12A3 CACNA1I SCN1B SCNN1A TRPM4 SCN7A GRIK4 SCN2B KCNJ1 SCNN1D SCN3B SCNN1G SCNN1B SCN4B CACNA1H KCNE1B |

Sheet 4

| Enrichment FDR | nGenes | Pathway Genes | Fold Enrichment | Pathway | URL | Genes |
| --- | --- | --- | --- | --- | --- | --- |
| 1.46E-06 | 4 | 37 | 102.6847 | Aldosterone-regulated sodium reabsorption | http://www.genome.jp/kegg-bin/show_pathway?hsa04960 | SCNN1A KCNJ1 SCNN1G SCNN1B |
| 0.000766 | 3 | 85 | 33.52353 | Taste transduction | http://www.genome.jp/kegg-bin/show_pathway?hsa04742 | SCNN1A SCNN1G SCNN1B |
| 0.008487 | 2 | 64 | 29.68229 | GnRH secretion | http://www.genome.jp/kegg-bin/show_pathway?hsa04929 | CACNA1I CACNA1H |
| 0.008487 | 2 | 65 | 29.22564 | Cortisol synthesis and secretion | http://www.genome.jp/kegg-bin/show_pathway?hsa04927 | CACNA1I CACNA1H |
| 0.000207 | 4 | 150 | 25.32889 | Adrenergic signaling in cardiomyocytes | http://www.genome.jp/kegg-bin/show_pathway?hsa04261 | SCN1B SCN7A SCN4B KCNE1B |
| 0.009898 | 2 | 76 | 24.99561 | Gastric acid secretion | http://www.genome.jp/kegg-bin/show_pathway?hsa04971 | CFTR KCNJ1 |
| 0.012659 | 2 | 97 | 19.58419 | Circadian entrainment | http://www.genome.jp/kegg-bin/show_pathway?hsa04713 | CACNA1I CACNA1H |
| 0.004335 | 3 | 169 | 16.86095 | Tight junction | http://www.genome.jp/kegg-bin/show_pathway?hsa04530 | CFTR NEDD4L NEDD4 |
| 0.023017 | 2 | 141 | 13.47281 | Ubiquitin mediated proteolysis | http://www.genome.jp/kegg-bin/show_pathway?hsa04120 | NEDD4L NEDD4 |
| 0.025077 | 2 | 155 | 12.25591 | Cushing syndrome | http://www.genome.jp/kegg-bin/show_pathway?hsa04934 | CACNA1I CACNA1H |
