# Supplementary material for: Differential expression of ion channel coding genes in the endometrium of women experiencing recurrent implantation failures
Source: Sci Rep. 2024 Aug 27;14:19822. doi: 10.1038/s41598-024-70778-9 (PMC11349755; doi:10.1038/s41598-024-70778-9)
Supplement: Supplementary file 4 — Supplementary Information 4. [file 41598_2024_70778_MOESM4_ESM.docx]

| **Significantly differentially methylated CpG sites between menstrual cycle phases.** | | | |  |  |  |
| --- | --- | --- | --- | --- | --- | --- |
| **CpG Site** | **chr** | **pos** | **logFC** | **P.Value** | **adj.P.Val** | **Annotated Gene** |
| cg13428066 | chr11 | 2677768 | 0.156414234 | 7.22E-08 | 3.25E-05 | KCNQ1;KCNQ1OT1;KCNQ1 |
| cg11700071 | chr11 | 2847778 | 0.170103301 | 1.06E-07 | 4.53E-05 | KCNQ1;KCNQ1 |
| cg13428066 | chr11 | 2677768 | 0.180396565 | 2.80E-06 | 0.002239691 | KCNQ1;KCNQ1OT1;KCNQ1 |
| cg23750514 | chr11 | 2790418 | 0.071853196 | 3.63E-06 | 0.000797348 | KCNQ1;KCNQ1 |
| cg26524638 | chr11 | 2481449 | -0.24108691 | 4.46E-06 | 0.018114809 | KCNQ1;KCNQ1 |
| cg11700071 | chr11 | 2847778 | 0.116236 | 7.49E-06 | 0.001477779 | KCNQ1;KCNQ1 |
| cg23267890 | chr11 | 2464970 | 0.123278782 | 3.06E-05 | 0.013169271 | KCNQ1 |
| cg23750514 | chr11 | 2790418 | 0.078423376 | 4.21E-05 | 0.007475131 | KCNQ1;KCNQ1 |
| cg17667688 | chr11 | 2495857 | -0.086308949 | 0.000281064 | 0.027626908 | KCNQ1;KCNQ1 |
|  |  |  |  |  |  |  |
| **Significantly differentially methylated regions between menstrual cycle phases.** | | | |  |  |  |
| **Chromosome** | **Start_bp** | **End_bp** | **Width (bp)** | **no.cpgs** | **min_smoothed_fdr** | **Stouffer** |
| chr11 | 2677768 | 2678450 | 683 | 2 | 1.60E-07 | 0.014803113 |
| chr11 | 2847258 | 2848492 | 1235 | 7 | 2.51E-06 | 0.158700779 |
| chr11 | 2790078 | 2791016 | 939 | 4 | 0.000589636 | 0.575529536 |
| chr11 | 2677768 | 2678450 | 683 | 2 | 2.72E-06 | 0.086476718 |
| chr11 | 2846932 | 2848492 | 1561 | 9 | 1.20E-08 | 0.294995974 |
