# Supplementary material for: Differential expression of ion channel coding genes in the endometrium of women experiencing recurrent implantation failures
Source: Sci Rep. 2024 Aug 27;14:19822. doi: 10.1038/s41598-024-70778-9 (PMC11349755; doi:10.1038/s41598-024-70778-9)
Supplement: Supplementary file 5 — Supplementary Information 5. [file 41598_2024_70778_MOESM5_ESM.docx]

| **Significantly differentially miRNA expression in GSE121219** | | | | | |  |
| --- | --- | --- | --- | --- | --- | --- |
| **ID** | **adj.P.Val** | **P.Value** | **t** | **B** | **logFC** | **miRNA_ID** |
| 145928 | 2.89E-06 | 2.35E-08 | 8.3142 | 9.3373 | 2.24377 | miR-344f-3p |
| 169167 | 2.99E-05 | 4.85E-07 | 6.9259 | 6.3288 | 1.96726 | hsa-miR-4451 |
| 168656 | 0.00012 | 2.52E-06 | 6.215 | 4.6906 | 2.19633 | hsa-miR-5002-3p |
| 169023 | 0.000537 | 2.09E-05 | 5.3353 | 2.5894 | 1.52759 | hsa-miR-4712-3p |
| 147923 | 0.00128 | 6.01E-05 | 4.9063 | 1.5451 | 2.07064 | hsa-miR-3142 |
| 169137 | 0.00133 | 6.45E-05 | 4.8779 | 1.4756 | 4.05301 | hsa-miR-4524b-5p |
| 46731 | 0.00185 | 0.000105 | 4.6812 | 0.995 | 2.10443 | hsa-miR-4657 |
| 145901 | 0.00307 | 0.00019 | 4.4436 | 0.4142 | 1.8788 | miR-4715-5p |
| 46207 | 0.00463 | 0.000316 | 4.2381 | -0.0865 | 1.96509 |  |
| 168769 | 0.00721 | 0.000562 | 4.0057 | -0.6497 | 1.58573 | hsa-miR-5002-5p |
| 46620 | 0.0102 | 0.000874 | 3.8267 | -1.0803 | 1.77915 |  |
| 10955 | 0.0107 | 0.00098 | 3.7803 | -1.1911 | 1.58161 | hsa-miR-148a-3p |
| 146001 | 0.0126 | 0.00119 | 3.7024 | -1.3766 | 2.18507 |  |
| 148217 | 0.0126 | 0.00121 | 3.6945 | -1.3954 | 1.50934 | hsa-miR-23c |
| 169009 | 0.0177 | 0.00193 | 3.5039 | -1.845 | 1.66275 | hsa-miR-548ap-5p/hsa-miR-548j |
| 169232 | 0.0195 | 0.00232 | 3.4282 | -2.0219 | 1.55487 | hsa-miR-3156-3p |
| 147667 | 0.0196 | 0.00235 | 3.4216 | -2.0372 | 1.57927 | hsa-miR-3182 |
| 42775 | 0.02 | 0.00248 | 3.4003 | -2.0867 | 1.67741 | miR-191 |
| 146220 | 0.0233 | 0.00314 | 3.3012 | -2.3155 | 1.69459 | miR-1251 |
| 147812 | 0.0245 | 0.00353 | 3.2527 | -2.4267 | 2.206 | miR-490-3p |
| 145992 | 0.0249 | 0.00366 | 3.2384 | -2.4595 | 1.54461 |  |
| 148430 | 0.0333 | 0.00562 | 3.0571 | -2.8689 | 1.83036 | hsa-miR-374c-5p |
| 169336 | 0.035 | 0.00614 | 3.0198 | -2.9519 | 1.54527 | hsa-miR-17-5p |
| 168995 | 0.0392 | 0.00732 | 2.9442 | -3.1191 | 1.52089 | hsa-miR-4791 |
| 145658 | 0.0477 | 0.00952 | 2.8308 | -3.366 | 2.03107 |  |
| 13150 | 0.0737 | 0.017 | 2.5751 | -3.9051 | 1.59388 |  |
| 146192 | 0.0739 | 0.0172 | 2.5705 | -3.9146 | 2.04454 |  |
| 169141 | 0.0744 | 0.0176 | 2.5589 | -3.9383 | 1.5226 | hsa-miR-4423-3p |
| 148470 | 0.0809 | 0.0197 | 2.5085 | -4.0411 | 1.64952 |  |
| 168994 | 0.105 | 0.0284 | 2.3398 | -4.3762 | 1.76514 | hsa-miR-3591-5p |
| 168915 | 0.115 | 0.0339 | 2.257 | -4.5356 | 1.68746 | hsa-miR-4780 |
| 147738 | 7.04E-11 | 1.14E-13 | -15.6148 | 21.1779 | -4.6663 | hsv2-miR-H20 |
| 147922 | 6.74E-09 | 2.19E-11 | -12.0729 | 16.1806 | -3.82431 |  |
| 146111 | 3.12E-07 | 1.52E-09 | -9.6834 | 12.0429 | -2.81827 | hsa-miR-767-5p |
| 148507 | 5.46E-07 | 3.55E-09 | -9.2458 | 11.2061 | -4.02502 |  |
| 42829 | 2.63E-05 | 2.87E-07 | -7.1578 | 6.8496 | -2.24185 | hsa-miR-127-3p |
| 169183 | 2.63E-05 | 3.21E-07 | -7.1093 | 6.7412 | -1.92002 | hsa-miR-4644 |
| 146158 | 2.63E-05 | 3.41E-07 | -7.0818 | 6.6798 | -2.09908 | hsa-miR-3202 |
| 148344 | 2.99E-05 | 4.81E-07 | -6.9295 | 6.337 | -1.95976 |  |
| 13138 | 5.74E-05 | 1.03E-06 | -6.5999 | 5.5852 | -2.09197 |  |
| 46336 | 0.00012 | 2.52E-06 | -6.2143 | 4.689 | -2.57273 | hsa-miR-1284 |
| 13388 | 0.000167 | 4.15E-06 | -6.0048 | 4.195 | -2.41844 | miR-582-3p |
| 42471 | 0.000167 | 4.3E-06 | -5.9901 | 4.1602 | -1.81759 | miR-3193 |
| 147920 | 0.000167 | 4.33E-06 | -5.9866 | 4.152 | -1.75848 | miR-5132 |
| 42927 | 0.000218 | 6.01E-06 | -5.85 | 3.8272 | -2.17549 | miR-4632 |
| 146210 | 0.00025 | 7.31E-06 | -5.7688 | 3.6334 | -2.00351 |  |
| 148678 | 0.000306 | 9.43E-06 | -5.6632 | 3.3805 | -2.69942 | hsa-miR-301a-5p |
| 169043 | 0.000334 | 1.08E-05 | -5.6055 | 3.2419 | -1.81042 | hsa-miR-4462 |
| 169025 | 0.000395 | 1.41E-05 | -5.4975 | 2.9816 | -2.67284 |  |
| 42960 | 0.000468 | 1.75E-05 | -5.409 | 2.7678 | -1.79104 |  |
| 169277 | 0.00063 | 2.66E-05 | -5.2374 | 2.3518 | -1.70092 |  |
| 28431 | 0.00063 | 2.76E-05 | -5.2219 | 2.3142 | -1.84164 |  |
| 27568 | 0.00104 | 4.71E-05 | -5.0056 | 1.7874 | -1.91506 | hsa-miR-744-5p |
| 17904 | 0.0014 | 7.06E-05 | -4.8413 | 1.3863 | -1.57521 |  |
| 148424 | 0.00176 | 9.71E-05 | -4.7129 | 1.0723 | -1.8333 |  |
| 17488 | 0.00304 | 0.000183 | -4.4585 | 0.4508 | -1.68902 |  |
| 145998 | 0.00357 | 0.000238 | -4.3527 | 0.1926 | -2.34528 |  |
| 42864 | 0.00721 | 0.000574 | -3.9972 | -0.6702 | -1.54815 |  |
| 146106 | 0.017 | 0.00176 | -3.5407 | -1.7588 | -1.79796 |  |
| 32812 | 0.02 | 0.00253 | -3.3911 | -2.108 | -1.57329 |  |
| 146043 | 0.0428 | 0.0081 | -2.901 | -3.2135 | -1.5052 |  |
| 17817 | 0.0684 | 0.0153 | -2.6214 | -3.8095 | -1.77731 |  |
| 146189 | 0.0988 | 0.0261 | -2.3786 | -4.3004 | -1.61878 | miR-1268 |
| 42570 | 0.13 | 0.0418 | -2.1565 | -4.7241 | -1.58526 | hsa-miR-194-3p |
